# Mechanism of hierarchical plasmonic biomaterials engineered through peptide-directed self-assembly

**DOI:** 10.1002/agt2.677

**Published:** 2024-10-10

**Authors:** Lubna Amer, Maurice Retout, Zhicheng Jin, Sumathi Kakanar, Jesse V. Jokerst

**Affiliations:** 1 Program in Materials Science and Engineering, University of California San Diego, San Diego, California, USA; 2 Aiiso Yufeng Li Family Department of Chemical and NanoEngineering, University of California San Diego, San Diego, California, USA; 3 Department of Radiology, University of California San Diego, San Diego, California, USA

**Keywords:** diffusion-limited aggregation, fractal, peptides, self-assembly, silver nanoparticles

## Abstract

Hierarchical plasmonic biomaterials constructed from small nanoparticles (NPs) that combine into larger micron-sized structures exhibit unique properties that can be harnessed for various applications. Using diffusion-limited aggregation (DLA) and defined peptide sequences, we developed fractal silver biomaterials with a Brownian tree structure. This method avoids complex redox chemistry and allows precise control of interparticle distance and material morphology through peptide design and concentration. Our systematic investigation revealed how peptide charge, length, and sequence impact biomaterial morphology, confirming that peptides act as bridging motifs between particles and induce coalescence. Characterization through spectroscopy and microscopy demonstrated that arginine-based peptides are optimal for fractal assembly based on both quantitative and qualitative measurements. Additionally, our study of diffusion behavior confirmed the effect of particle size, temperature, and medium viscosity on nanoparticle mobility. This work also provides insights into the facet distribution in silver NPs and their assembly mechanisms, offering potential advancements in the design of materials for medical, environmental, and electronic applications.

## 1 ∣ INTRODUCTION

Nanomaterials have garnered significant attention for their exceptional physical and chemical properties.^[1-3]^ Plasmonic metallic nanoparticles, such as gold (AuNPs) and silver (AgNPs), are particularly fascinating due to their tunable morphology, high optical absorption/scatter, and facile synthesis. These NPs exhibit a localized surface plasmon resonance (LSPR) band—a phenomenon caused by the collective oscillation of electrons in response to light.^[4-6]^ This LSPR band, and thus, their electronic and optical properties, can be tuned by altering the size, shape, and surface chemistry of the NPs.^[7-9]^ For example, spherical gold or silver NPs have LSPR bands in the visible region, and anisotropic NPs can have bands in the near-infrared (NIR)-I region.^[10-13]^ The unique colorimetric signal mechanism is particularly useful in sensing applications, such as point-of-care sensors,^[14-17]^ while the NIR-I region is known for its reduced light scattering and absorption by biological tissues, allowing deeper tissue penetration, ideal for biomedical imaging.^[16]^ AuNPs are more common because of their high sensitivity and biocompatibility. However, AgNPs, with their higher molar absorption coefficient, exhibit multiple color changes and span the entire visible range, making them useful in medical and environmental contexts. The versatility and multifunctionality of plasmonic metallic NPs continue to drive innovation and discovery across multiple disciplines.^[18-20]^

The hierarchical assembly of NPs is an extension of this phenomenon and offers properties and functionalities beyond those of individual NPs such as increased surface area,^[21,22]^ improved signal amplification,^[23,24]^ and synergistic interactions that mimic biomolecules.^[25-27]^ This assembly involves NPs organizing into larger, micronsized, complex structures, often through self-assembly or directed assembly techniques.^[28-31]^ Typically, these larger structures are built through solvent evaporation,^[32,33]^ layer-by-layer deposition,^[34]^ or templates and external fields.^[35]^ These hierarchical structures are particularly advantageous in applications such as catalysis, sensing, and photonics, where the collective behavior of the assembled NPs can lead to improved performance.^[36]^ For instance, De Fazio et al. reported DNA-mediated gold nanoparticle fractal assemblies that exhibit enhanced stability and light-induced reversibility of the assembly, thus altering their LSPR and optical characteristics.^[37]^ The ability to precisely control the arrangement and interaction of NPs opens up new avenues for designing advanced materials with tailored properties. However, current reported techniques for hierarchical assembly of NPs require complex synthesis procedures and redox chemistry, making them less accessible for widespread applications.

We previously showed that we can build peptide-induced fractal silver structures by diffusion-limited aggregation (DLA).^[38]^ However, this prior work had an incomplete explanation of the precise mechanisms driving the self-assembly process and how different parameters influence the final structure. In this context, we now report a mechanistic study on peptide-induced fractal silver structures built by DLA. Here, the peptide facilitates nanoparticle assembly by modifying the particle surface properties and prompting spontaneous coalescence.^[39]^ As the aggregates grow, they block areas between aggregated particles, creating a fractal structure or Brownian tree.^[40]^ These dendritic structures result from the self-assembly of molecules driven by free energy minimization of the system, primarily through diffusion.^[41]^ By relying on controlled coalescence, we can control interparticle distance and material morphology using only peptide design and concentration, resulting in scalable and tunable biomaterials. We constructed these fractal structures by incubating ~20 nm silver nanospheres with certain short peptides (3–13 residues). Based on this, we offer a coherent mechanistic understanding of how peptide charge, peptide size, and amino acid sequence impact biomaterial morphology. We characterized the structures using spectroscopy and microscopy and employed a thorough peptide library to outline the noncovalent interactions and their effect on morphology. Finally, we determined that specific electrostatic interactions between the peptide bridging motif, and the nanoparticle surface ligand are the key parameters for forming fractal structures with high fractal dimensions.

## 2 ∣ RESULTS AND DISCUSSION

### 2.1 ∣ Bridging motif and nanoparticle assembly size

**SCHEME 1:**
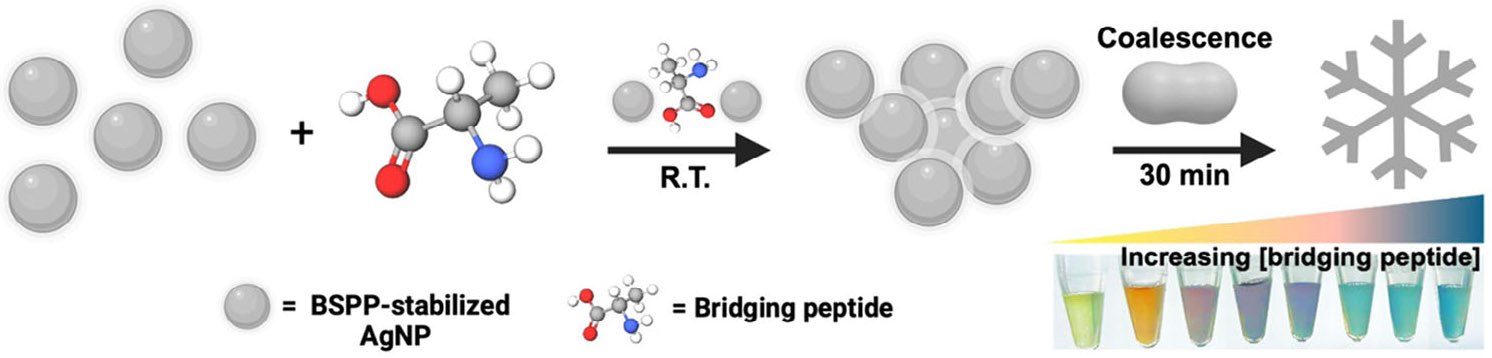
Illustration of the peptide-induced assembly of nanoscale bis(p-sulfonatophenyl)phenylphosphine dihydrate dipotassium-silver nanoparticles (BSPP-AgNPs) and their coalescence into micron-sized fractal structures.

In our previous work, we showed that “bridging” peptides with at least two repetitions of amino acids (i.e., arginine, histidine, or phenylalanine) can interact with bis(p-sulfonatophenyl)phenylphosphine dihydrate dipotassium (BSPP)-coated AgNPs (BSPP-AgNPs) via electrostatic, π-π, or hydrophobic interactions.^[38]^ On this basis, we determined that electrostatic interactions, specifically those between arginine (R)-based peptides and BSPP, were optimal for fractal assembly and timely color change (Scheme 1). Optimal assembly was defined by a minimum number of residual (free) particles and maximum color change as measured by dynamic light and optical spectroscopy, respectively. The mode of interaction was validated by adjusting the pH, and a decrease in assembly intensity was observed as the pH approached the pKa of the arginine side chain (~12).

**FIGURE 1:**
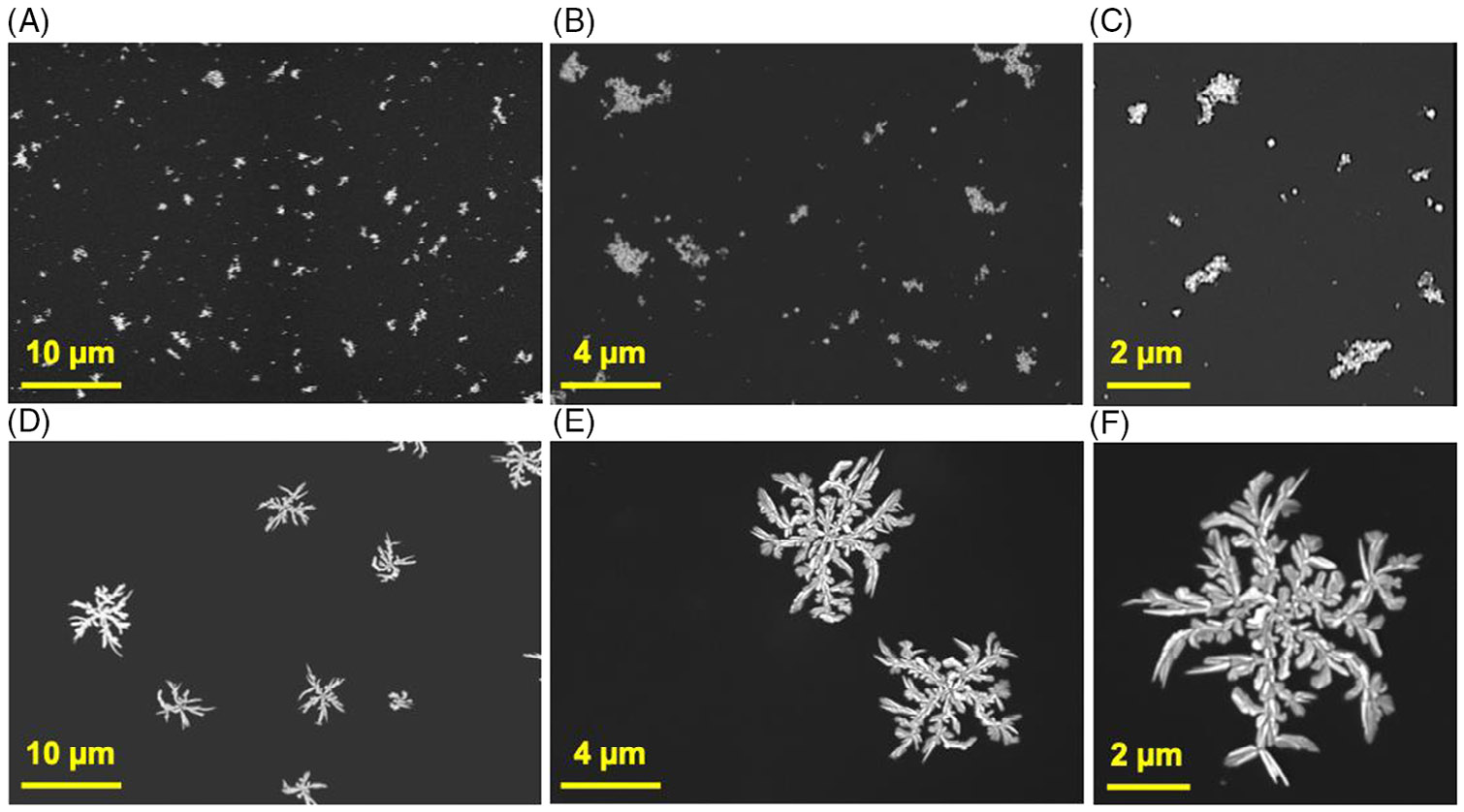
Peptide-mediated fractal assembly of bis(p-sulfonatophenyl)phenylphosphine dihydrate dipotassium-silver nanoparticles (BSPP-AgNPs) and changes in their morphology. (A–C) Scanning electron microscopy (SEM) images of BSPP-AgNPs incubated with negative control peptide, threonine-serine-glycine (TSG) (1 μM), showing dispersed nanoparticles at increasing magnifications. (D–F) SEM images of BSPP-AgNPs after a 30-min incubation at room temperature with 1 μM arginine-arginine-lysine (RRK) showing complete coalescence and reshaping of nanoparticles.

We first replicated this to demonstrate the highly structured assembly of BSPP-AgNPs via DLA using arginine- arginine-lysine (RRK) as a lead candidate. BSPP-AgNPs were synthesized via a two-step seed-mediated growth protocol (Figure S1) and characterized via spectroscopy (ultraviolet-visible [UV–vis], dynamic light scattering [DLS], and Fourier-transform infrared), microscopy (transmission electron), and multi-laser nanoparticle tracking analysis (M-NTA) (Figures S2-S6). The peptides were synthesized via standard solid-phase Fmoc synthesis, purified using high-performance liquid chromatography (HPLC), and characterized using electron spray ionization mass spectrometry (ESI-MS) (see Methods; Figure S7).^[31,42-44]^ An uncharged peptide, threonine-serine-glycine (TSG), was used as a negative control peptide due to its similarity in molecular weight and length to the lead candidate, RRK. We have shown that TSG does not influence particle assembly even at millimolar concentrations (Figure 1A-C).^[38,45]^ Conversely, RRK induced the coalescence of the particles and complete reshaping (Figure 1D-F and Figure S8).

Material stability was monitored over a 17-h period, with the plateau occurring at the 30-minute mark (Figure S9). Fractal growth was also monitored over time (Figure S10).^[38]^ Importantly, no decrease in assembly intensity or deformation of material morphology was observed during this time frame, confirming the stability of the fractal structures when preserved from direct light.^[46,47]^ To examine the consequences of 254 nm light exposure at 7.22 mW/cm^2^,we imaged the structures via transmission electron microscopy (TEM) after 20 min of consistent illumination. The images revealed significant material degradation, with the particles coalescing into round forms instead of maintaining their original fractal structures (Figure S11). This indicates a clear morphological change due to the light exposure. We next quantified RRK concentration using a Bradford assay to determine the effects on peptide adhesion (Figure S12).^[48,49]^ This assay uses a Coomassie Brilliant Blue dye that undergoes a shift in its absorbance maximum from 465 to 595 nm upon binding to amino acids, particularly arginine residues, which are abundant in RRK. We thus determined that light exposure resulted in desorption of RRK, and a 4 times increase in the peptide concentration in solution. This substantial increase in RRK concentration suggests that the stability of the peptide-nanoparticle interface is compromised, highlighting the weak non-covalent interactions responsible for fractal assembly.

Finally, fractal reshaping was validated using reversibility experiments. Here, silver pellets freshly clustered with 1 μM RRK could not be redispersed in any of the surfactants or solvents used (Figure S13).^[50]^ This irreversibility suggests that the particles formed diffusion-limited aggregates and not reaction-limited aggregates, making it more difficult for surfactants or solvents to effectively redisperse them.^[51]^ This irreversibility is also comparable to particles that have undergone chemical reactions that cause them to bind together covalently,^[50]^ further confirming that the NPs have undergone coalescence to form new structures.

### 2.2 ∣ Diffusion behavior of AgNPs

**FIGURE 2:**
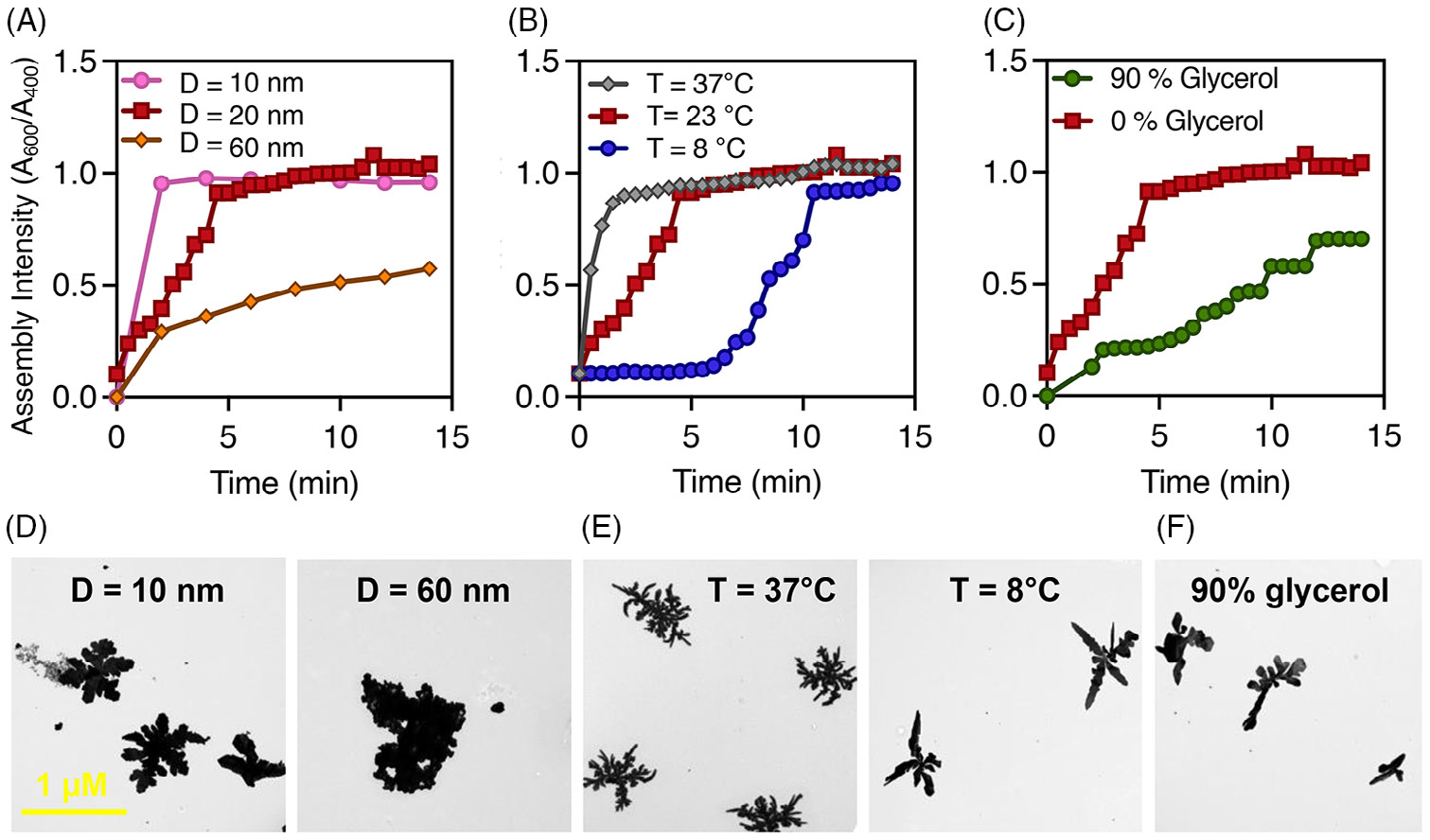
Diffusion behavior as described by the Stokes–Einstein equation. (A) Evolution of the assembly intensity $\left({\text{Abs.}}_{600\;\text{nm}}∕{\text{Abs.}}_{400\;\text{nm}}\right)$ as a function of time for varying nanoparticle sizes after the introduction of 1 μM arginine-arginine-lysine (RRK) peptide. This ratio corresponds to spherical bis(p-sulfonatophenyl)phenylphosphine dihydrate dipotassium-silver nanoparticles (BSPP-AgNPs) (Abs. at 400 nm) and larger structures (Abs. at 600 nm). Here, a 50% decrease in size caused the diffusion coefficient to increase two-fold, which in turn led to a faster assembly of BSPP-AgNPs by 2 min. Conversely, a three-fold increase in size leads to a 33% decrease in the diffusion coefficient and a significant increase in assembly time (*p* < 0.05). (B) Evolution of the assembly intensity $\left({\text{Abs.}}_{600\;\text{nm}}∕{\text{Abs.}}_{400\;\text{nm}}\right)$ as a function of time at varying temperatures after introduction of 1 μM RRK peptide. Here, an incubator was used to heat the peptide and particle solutions before mixing. Similarly, an ice bath was used to cool both solutions to 8°C before mixing. The results show that a 65% cooler temperature decreased the diffusion coefficient by 5%, which in turn led to a slower assembly of BSPP-AgNPs by 8 min. Conversely, a 61% increase in temperature lead to a 5% increase in the diffusion coefficient and an increase in assembly time by 3 min. (C) Evolution of the assembly intensity $\left({\text{Abs.}}_{600\;\text{nm}}∕{\text{Abs.}}_{400\;\text{nm}}\right)$ as a function of time with various solvent viscosities: water (0% glycerol) and 90% glycerol after the introduction of 1 μM RRK peptide. The viscosity of the glycerol solution is over 1000-fold higher than that of water, causing a decrease in the diffusion coefficient to 0.5% compared to water and a much slower assembly time. (D) Corresponding transmission electron microscopy (TEM) images showing the different structures obtained for BSPP-AgNPs after reacting with RRK for 30 min with increasing particle size (left to right). (E) Corresponding TEM images showing the different structures obtained for BSPP-AgNPs after reacting with RRK for 30 min at decreasing temperatures (left to right). (F) Corresponding TEM image showing the different structures obtained for BSPP-AgNPs after reacting with RRK for 30 min in glycerol. Note that the glycerol TEM grid was manually dried by blotting.

Next, we systematically investigated the diffusion behavior of the BSPP-AgNPs to demonstrate the underlying dynamics of RRK-mediated DLA. The LSPR band of BSPP-AgNPs deforms strongly upon the addition of RRK with decreased 400 nm absorbance and an increase at 600 nm.^[38]^ We thus monitored the deformation of the UV–vis spectrum over time and defined assembly intensity as ${\text{Abs.}}_{600\;\text{nm}}∕{\text{Abs.}}_{400\;\text{nm}}$. Using the Stokes–Einstein equation (Equation (1)), we calculated the diffusion coefficients $\left(d_{H}\right)$ of BSPP-AgNPs of varying diameters (Figure 2A), under different thermal conditions (Figure 2B), and in a viscous environment (Figure 2C and Table 1).


(1) $$d_{H}=\frac{k_{B}T}{3\pi \eta D}$$

Here, $d_{H}$ represents the diffusion coefficient; $k_{B}$ is the Boltzmann constant; T is the absolute temperature; η is the dynamic viscosity; and D is the particle diameter.

Our results demonstrate a clear inverse relationship between the particle diameter and the diffusion coefficient, confirming that smaller particles diffuse more rapidly. José-Yacamán et al. claim that the driving force for the coalescence of two NPs is the surface energy reduction: the surface area of the incoming nanoparticle is less than that of the aggregate.^[52]^ Thus, as the nanoparticle size (D) decreases, the driving force (Helmholtz surface energy per unit area) increases, causing an increase in the diffusive behavior. This enhanced diffusion leads to a higher probability of encountering other particles, facilitating coalescence. Consequently, smaller particles are more prone to DLA, where the rate of aggregation is controlled by the rate at which particles diffuse together.^[53]^ As smaller particles readily diffuse and coalesce, they continue to form fractal structures, illustrating the critical role of diffusion in the assembly process.

Additionally, we observed that increasing or decreasing the reaction temperature by 65% resulted in a proportional increase or decrease in the diffusion coefficients. This trend highlights the temperature dependence of nanoparticle mobility. Conversely, a 1000-fold reduction in the diffusion coefficient was noted in the highly viscous 90% glycerol solution compared to water, underscoring the role of medium viscosity in impeding particle diffusion, as glycerol’s higher viscosity provides greater resistance to particle movement compared to water. These experimental observations are consistent with the principles of DLA, where particle aggregation and cluster formation are predominantly influenced by diffusion processes.^[41,54]^ Interestingly, a fractal shape is still observed after the decrease in particle size (Figure 2D), change in solution temperature (Figure 2E), or increase in viscosity (Figure 2F). We thus confirmed that, while the time scale can be increased or reduced, branched structures can be formed as a result of the arginine-based peptide incubation. This data further highlights the importance of the assembly interaction between the ligand and peptide and unequivocally shows that the system is a function of DLA.

### 2.3 ∣ Facet distribution in AgNPs and fractal structures

**FIGURE 3:**
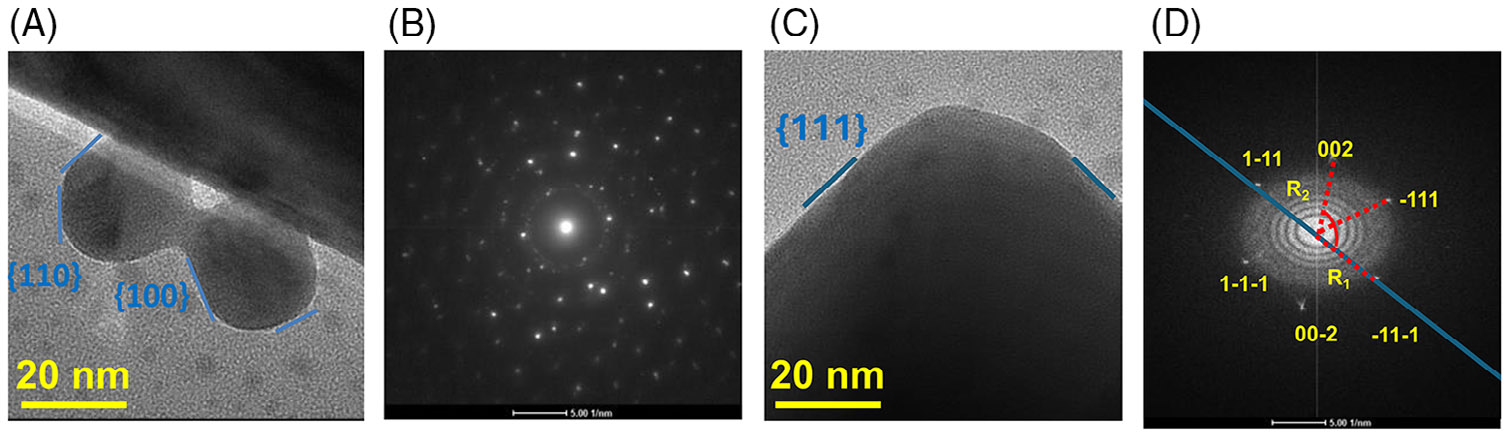
Facet distribution in silver nanoparticles and fractal structures. (A) Neck formation at the contacting surfaces of the individual bis(p-sulfonatophenyl)phenylphosphine dihydrate dipotassium-silver nanoparticles (BSPP-AgNPs). This shows the different crystal structures and facet distribution derived from the (B) diffraction pattern of silver nanoparticles. (C) Facet distribution showing newly formed “relaxed” structure derived from the (D) diffraction pattern for the fractal structure edge. The scale bars in (A) and (C) represent 20 nm and 5.00 1/nm in (B) and (D).

The diffraction patterns were collected to identify the crystalline structures during RRK-mediated particle assembly. These patterns function as precise “fingerprints”, enabling us to determine whether a sample is single-crystal, polycrystalline, or amorphous.^[55,56]^ This facilitates accurate measurement of lattice parameters and identification of multiple phases.^[57,58]^ The specific distribution of these facets is significantly influenced by the nanoparticle size, shape, and synthesis method. We thus sought to confirm the facet distribution during different stages of the fractal assembly process. We observed that the dispersed AgNPs exhibited a mix of facets, including {111}, {100}, and {110} (Figure 3A,B). This is likely because of the interplay between thermodynamic stability and kinetic growth processes during nanoparticle formation.^[59]^

Notably, when the NPs aggregate, coalesce and form fractal structures, the fractal edges, and tips are predominantly composed of {111} facets (Figure 3C,D). In the context of DLA, particles aggregate through random collisions and attachment processes, leading to the formation of the observed complex dendritic structures. As the fractal structure grows and evolves, surface atoms undergo reconstruction and annealing processes, resulting in the minimization of surface energy and stabilization of specific facets.^[51,52]^ Growth conditions or the dynamics of particle merging are likely responsible for promoting the formation of {111} facets because of their higher growth rates compared to {100} or {110} facets.^[59]^ These kinetic factors can override the thermodynamic preference for lower energy facets, resulting in a greater prevalence of {111} facets during rapid growth and aggregation.^[60]^ It is also possible that the peptides act as surfactants. For FCC materials such as silver, surfactant binding is cited to be much stronger on specific facets (e.g., {111} facet for FCC crystals) and is thought to result in 2D nanostructures with the lowest surface energy facet, {111}.^[51,61]^ This also confirms that silver NPs can behave as “soft” particles and undergo spontaneous coalescence under ambient conditions after modification of their surface properties via charge neutralization and ligand desorption.^[62,63]^

### 2.4 ∣ Different mechanisms of peptide-induced self-assembly

We next investigated the interactions between the peptide and particle surface ligand. We have previously shown that repeat units of RRK, for example, ${\left(\text{RRK}\right)}_{n}$ where n=1, 2 and 3 (Table 2; p1–p3), lead to increased assembly intensity relative to monomeric RRK at isomolar concentration due to increased electrostatic attractions.^[38]^ We now sought to investigate the effects of peptide charge and length on fractal structure and determine whether repeat units of other peptides will do the same for electrostatic/π-π forces. Lysine, histidine and phenylalanine peptides were selected as representative peptides of electrostatic, electrostatic/π-π, and π-π interactions, respectively (Table 2; p4–p12).

**FIGURE 4:**
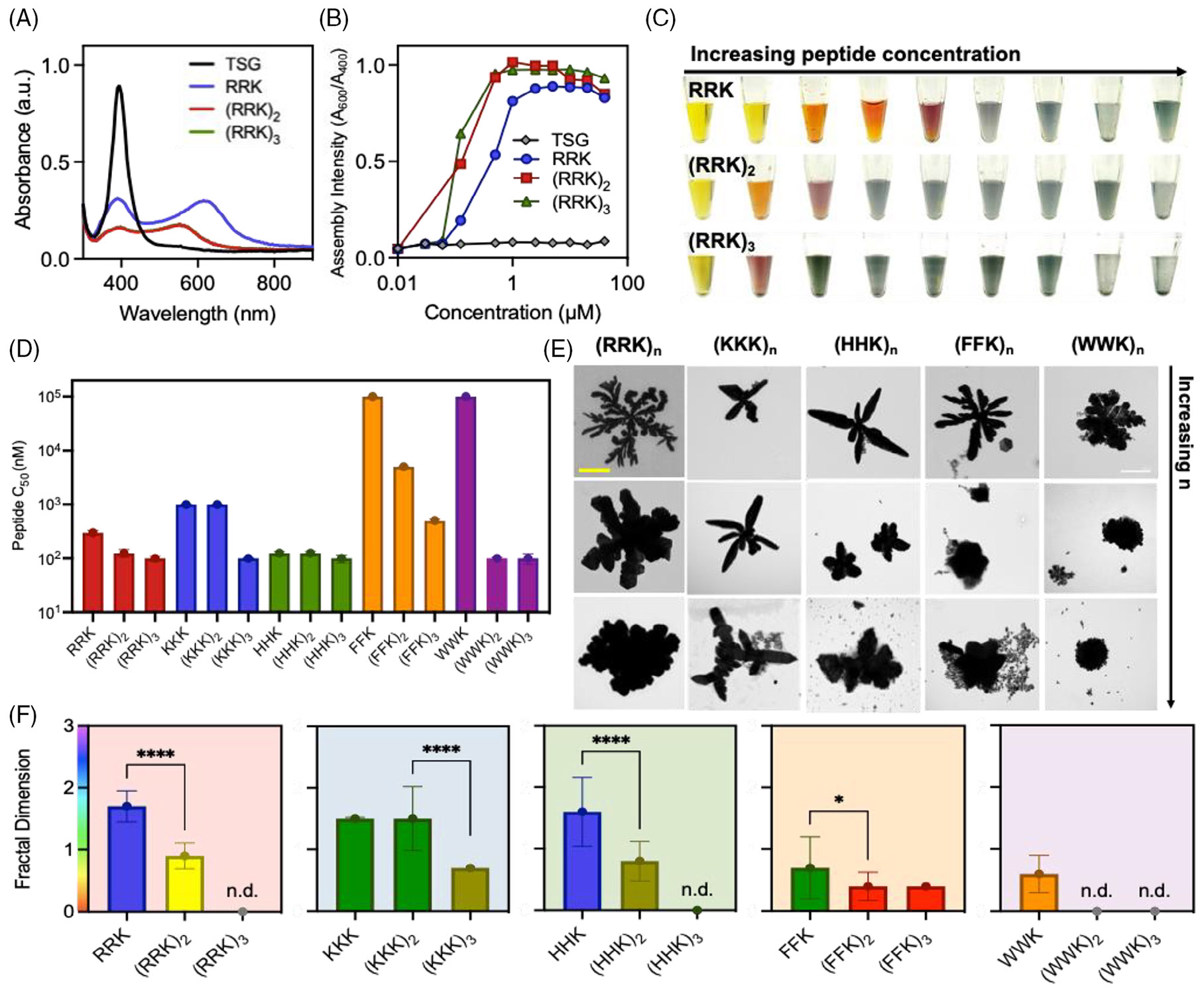
Non-covalent interactions of peptide-induced self-assembly. (A) Ultraviolet-visible (UV–vis) spectra of bis(p-sulfonatophenyl)phenylphosphine dihydrate dipotassium-silver nanoparticles (BSPP-AgNPs) after 30 min of reaction with RRK (blue), (RRK)_2_ (red), and (RRK)_3_ (green). Threonine-serine-glycine (TSG) (black) was used as a neutral amino acid peptide (negative control). (B) Evolution of the assembly intensity $\left({\text{Abs.}}_{600\;\text{nm}}∕{\text{Abs.}}_{400\;\text{nm}}\right)$ as a function of peptide concentration. This ratio corresponds to spherical BSPP-AgNPs (Abs. at 400 nm) and larger structures (Abs. at 600 nm). (C) $C_{50}$ values, defined as the peptide concentration causing a 50% change in the assembly intensity: ${\text{Abs.}}_{600\;\text{nm}}∕{\text{Abs.}}_{400\;\text{nm}}$ ratio of the peptides depicted in Table 2. Error bars represent standard deviation (*n* = 9). (D) Colorimetric evolution of an example peptide family, ${\left(\text{RRK}\right)}_{n}$, over increasing peptide concentrations from 0 to 100 μM (left to right). (E) Representative transmission electron microscopy (TEM) images showing the different structures obtained for BSPP-AgNPs after reacting with each peptide for 30 min. Increased repetitions of the bridging motif (n=1,2,and3) are presented from top to bottom. The scale bar represents 1 μm and all images were taken at the same magnification. (F) Fractal dimension defining the complexity of the average fractal structure. ROYGBV color scheme of the bars represent fractal complexity on a scale from 0 to 3. Error bars represent standard deviation from 24 individually imaged structures. Significance is determined when α<0.05: **** (*p* < 0.0001); * (*p* < 0.05). Not determined: n.d., is denoted when no fractal structure is found by the FracLac extension.

The dynamic process of the nanoparticle assembly was systematically investigated by monitoring the deformation of the UV–vis spectrum based on the peak shift (Figure 4A). A titration was conducted from 0 to 100 μM to determine the effect of concentration on assembly intensity (Figure 4B). We thus determined that an increase in the number of repetitions of the bridging motif resulted in an earlier redshift at lower peptide concentrations: ${\left(\text{XXK}\right)}_{3}>{\left(\text{XXK}\right)}_{2}>\text{XXK}$ (Figure S14). Colorimetric evolution followed, where a more sudden transition from yellow to blue was observed at higher n values (Figure 4C). These results were consistent across all peptides. We thus defined $C_{50}$ as the peptide concentration causing a 50% change in the assembly intensity and used this metric to compare the peptides’ assembly properties (Figure 4D). We concluded that the optical response was proportional to the increase in the number of repeat units for all peptides such that the $C_{50}$ decreased by nearly threefold (33%) between XXK and ${\left(\text{XXK}\right)}_{2}$ and by half (49%) for ${\left(\text{XXK}\right)}_{3}$.

Interestingly, the $C_{50}$ values of the lysine-based peptides were on average, four times greater than those of the arginine-based peptides. This difference is likely due to the guanidine group in arginine, which allows interactions in three possible directions with the anionic counterparts through its three asymmetrical nitrogen atoms. This contrasts with the straight aliphatic chain of lysine, which ends in a primary amine. Arginine also has a slightly higher pKa of 12.5, so it may be slightly more positively charged than lysine (pKa = 10) at the same physiological pH (Figure S15). Based on the $C_{50}$ values, we also concluded that the effect of ${\left(\text{HHK}\right)}_{n}$ closely mimics that of ${\left(\text{RRK}\right)}_{n}$ (81% $C_{50}$ similarity). This is likely due to a combination of both electrostatic and hydrophobic interactions.^[38]^ Conversely, phenylalanine-based peptides expressing primarily π-π interactions had a 10,000-fold increase in $C_{50}$, suggesting that BSPP is significantly less sensitive to π-π interactions. This increase confirms that amino acids with aromatic side groups can lead to self-assembly of the BSPP-AgNPs, albeit at higher concentrations. To affirm this conclusion, we also designed tryptophan (W) based peptides (Table 2; p13–p15). We determined that peptide sequences containing W residues induce the assembly of BSPP-AgNPs at lower $C_{50}$ values than F because tryptophan is more hydrophobic than the phenylalanine residues shown previously to induce assembly.^[64,65]^ We thus concluded that monomeric sequences containing repetitions composed of K, H, F and W can also induce coalescence and fractal formation under suitable conditions.

The structures were also analyzed via TEM. The TEM images confirmed that polymeric structures (n=2,3) had an increase in aggregation density and a breakdown of the fractal structure (Figure 4E). Here, aggregation density is defined as a scarcity of free particles and coalescence throughout the sample as shown across the TEM grid (Figures S16-S30). It is likely that the increase in repetitions led to increased nanoparticle coalescence relative to monomeric XXK at isomolar concentrations due to increased interactions. This data suggests an exponential relationship between the number of repetitions and the aggregation increase. This is indicative of multivalent effects: as the number of peptide repetitions increases, the interactions among peptides might enhance beyond simple summation, leading to more complex coalescence behavior and larger aggregate sizes. The hydrodynamic diameter $\left(D_{H}\right)$ distributions were also obtained from DLS to show an increase in size (~1000 nm) proportional to the increase in bridging peptide repetitions (Figure S31).

Next, the fractal dimension was calculated to quantitatively assess the assemblies observed via TEM (Figure 4F). Here, the FracLac plugin in ImageJ was used for fractal analysis (see Methods; Figure S32).^[66,67]^ The fractal dimension was defined to measure how complex each fractal pattern is on a scale from zero to three. The plugin employs various methods, including box counting, to provide a quantitative measure of the complexity, self-similarity, and space-filling properties of fractals, enabling comparisons between different fractal patterns and facilitating their analysis.^[53,68,69]^ The maximum fractal dimension for DLA typically approaches 1.71 in two dimensions.^[70]^ This is because DLA is a stochastic growth process where particles move randomly. Thus, the maximum value reflects the complex and self-similar nature of the DLA clusters, where an equilibrium state is observed. The branching structures formed by the aggregation of particles achieve a peak level of complexity where further growth does not significantly increase the fractal dimension. Based on this quantitative measure, monomeric peptides exhibited the highest fractal dimension (~1.7): the fractal dimension decreased with an increase in bridging motifs. Importantly, if the dimeric peptide, that is, ${\left(\text{XXK}\right)}_{2}$ was able to form fractal structures, as defined by an output fractal dimension, then the decrease in fractal dimension was 52%. If the trimeric peptide was able to do so, the fractal dimension again decreased by half. This is likely due to the increased interparticle interactions in all directions, which causes less structural heterogeneity and therefore a decrease in the fractal dimension at the investigated concentrations. Sample images are presented in Figure S33 along with a statistical analysis of significance.

### 2.5 ∣ Effects of nonbridging amino acid spacers on self-assembly

**FIGURE 5:**
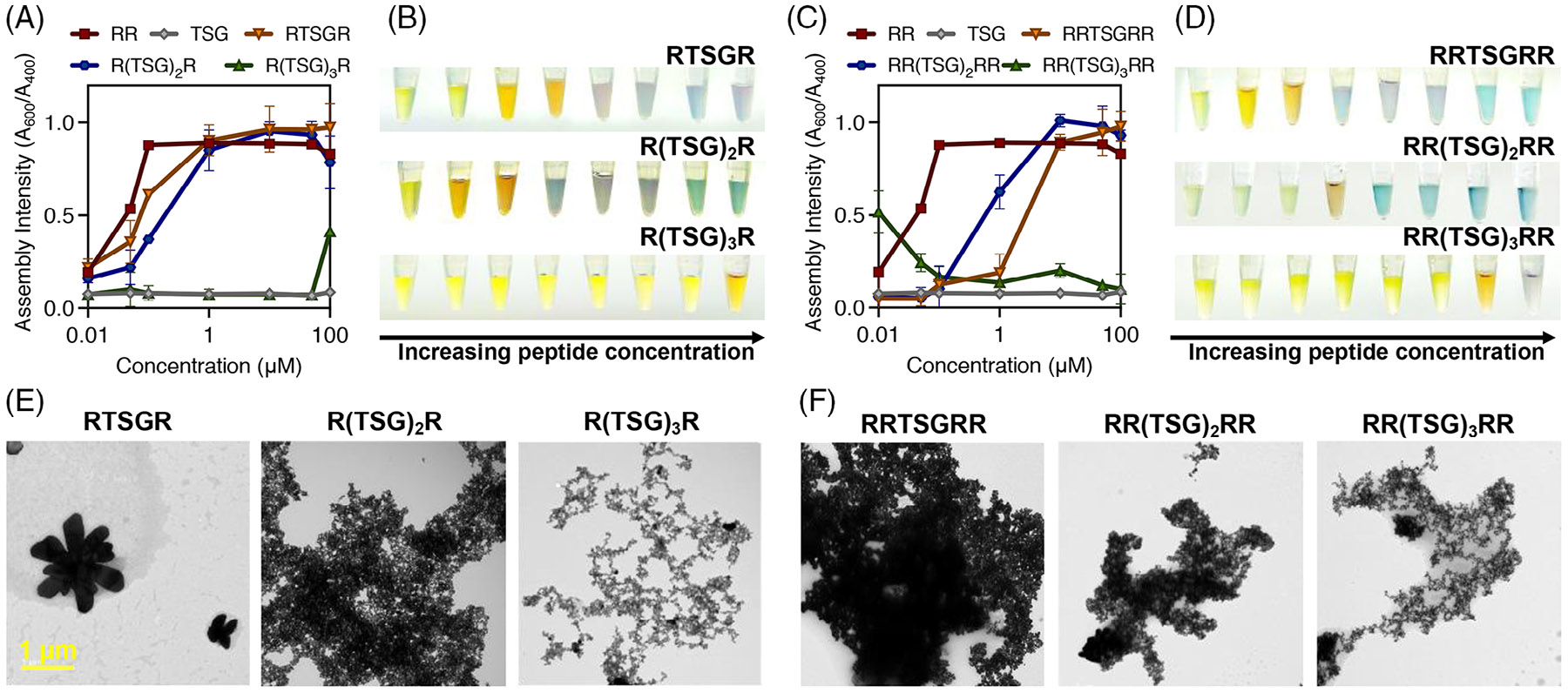
Peptide-mediated assembly with a nonbridging amino acid spacer. (A) Evolution of the assembly intensity $\left({\text{Abs.}}_{600\;\text{nm}}∕{\text{Abs.}}_{400\;\text{nm}}\right)$ as a function of the peptide concentration and (B) the corresponding images from 0 nM to 100 μM. RTSGR (orange), R(TSG)_2_R (blue), and R(TSG)_3_ R (green). Threonine-serine-glycine (TSG) (black) was used as a nonbridging amino acid (negative control), and RR (red) was used as a positive control. (C) Evolution of the assembly intensity $\left({\text{Abs.}}_{600\;\text{nm}}∕{\text{Abs.}}_{400\;\text{nm}}\right)$ as a function of the peptide concentration of charge-dense peptides with nonbridging amino acid spacers and (D) the corresponding images from 0 nM to 100 μM. RRTSGRR (orange), $\text{RR}{\left(\text{TSG}\right)}_{2}\text{RR}$ (blue), and $\text{RR}{\left(\text{TSG}\right)}_{3}\text{RR}$ (green). TSG (black) was used as a nonbridging amino acid (negative control), and RR (red) was used as a positive control. (E, F) Transmission electron microscopy (TEM) images showing the different structures obtained for bis(p-sulfonatophenyl)phenylphosphine dihydrate dipotassium-silver nanoparticles (BSPP-AgNPs) after reacting for 30 min with increased repetitions of the nonbridging spacer from left to right. The scale bar represents 1 μm and all images were taken at the same magnification.

We next investigated the effects of nonbridging amino acid spacers on self-assembly by interspacing the negative control, TSG, between two arginine units (Table 2; p16–p18). Our aim was to discern whether the assembly was more driven by total charge or charge density. The results revealed that increasing the length of the ${\left(\text{TSG}\right)}_{n}$ spacer reduces aggregation density, particularly notable after three repeats (Figure 5A). Interestingly, RTSGR behaved similarly to RR, suggesting that one TSG spacer has negligible effects on assembly behavior. Conversely, $R{\left(\text{TSG}\right)}_{3}R$ caused a 92% decrease in assembly intensity compared to RTSGR at isomolar concentrations. This reduction is also observable optically, where the samples remain yellow even at high concentrations of $R{\left(\text{TSG}\right)}_{3}R$ (Figure 5B). This confirms that increasing the distance between two arginine units hinders assembly. We next designed charge-dense peptides: $\text{RR}{\left(\text{TSG}\right)}_{n}\text{RR}$ (Table 2; p19-p21) to verify this conclusion. This data suggests that peptides with two adjacent RR units exhibit a maximum charge density beyond which additional charges do not enhance aggregation (Figure 5C,D). We thus concluded that exceeding ten amino acid residues, such as in $R{\left(\text{TSG}\right)}_{3}R$ and $\text{RR}{\left(\text{TSG}\right)}_{3}\text{RR}$, leads to no assembly due to insufficient coupling of adjacent NPs.

This finding is reiterated through microscopy (Figure 5E,F). TEM images show physical spacing between individual NPs for longer peptides, with expanded magnifications available in the supplemental information (Figures S34-S36). However, these structures are limited to random assembly, and fractal structures are not observed (Figures S37-S39). DLS data further demonstrate that more residual NPs are present with longer spacers, indicating that spacer length critically influences the self-assembly process, likely because particles are too distant for effective coalescence (Figure S40). Structural modeling supports the idea that an increase in amino acid residues increases the length which may contribute to decreased aggregation density (Figure S41). It is also probable that the Brownian motion of the peptide can influence assembly.^[71,72]^ That is, longer peptides are less likely to effectively collide and interact with NPs. This slower movement diminishes the frequency and energy of collisions necessary for coalescence, making the process less efficient. This supports our hypothesis that peptides act as bridges and that beyond a certain charge density threshold, further increases do not enhance assembly, highlighting a critical limit for charge-mediated interactions in peptide assembly.

## 3 ∣ CONCLUSION

This study demonstrated the efficacy of using DLA and defined peptide sequences to construct hierarchical silver biomaterials with fractal snowflake-like designs. Our findings demonstrate that the assembly of hierarchical plasmonic biomaterials is driven primarily by electrostatic interactions between the nanoparticle surface, which is coated with BSPP, and arginine-based peptides. Among the tested peptides, those containing arginine (R), specifically the ${\left(\text{RRK}\right)}_{n}$ sequences, were found to be the most effective at promoting fractal assembly with high fractal dimensions due to their high charge density and optimal electrostatic interactions. The repeating units of the bridging peptides significantly affect the fractal dimensions, with increased repetitions leading to more dense structures. However, the highest fractal dimension was observed with monomeric peptides, suggesting that while additional repeats enhance aggregation, they can also reduce structural heterogeneity. We thus addressed the challenge of simplifying the synthesis process while maintaining control over the hierarchical assembly of plasmonic biomaterials by leveraging peptide-directed self-assembly and DLA. These insights provide a deeper understanding of self-assembly mechanisms and offer practical approaches for designing advanced plasmonic biomaterials with controlled morphology and functionalities that can be applied to biosensing. This work does have some limitations: Most importantly, the mechanism is not completely elucidated including the exact role of intermolecular interactions versus redox chemistry in dendrite formation. We are doing careful yet time-consuming XPS studies to further understand the role of redox chemistry, and these results will be published in future work. We also describe a reliance on specific peptide sequences and a BSPP coating, which may limit the generalizability of the findings to other nanoparticle systems or biomaterial designs. Thus, future work aims to investigate the role of BSPP via alternative ligands.

## 4 ∣ EXPERIMENTAL SECTION

### 4.1 ∣ Synthesis of BSPP-AgNPs

BSPP-coated AgNPs (BSPP-AgNPs) were synthesized using a two-step procedure. First, silver seeds were produced by the reduction of silver nitrate (0.1 mM, 6 mL) with sodium boro-hydride (0.1 M, 60 μL) in a glass vial under magnetic stirring for 16 h. The seeds were then grown into NPs by adding sodium ascorbate (15 mM, 400 μL), BSPP (5 mM, 200 μL), dropwise silver nitrate (1 mM, 4 mL), and then BSPP again (5 mM, 400 μL), and the resulting solution was stirred for 48 h at room temperature. For alternative sizes, we adjusted the timeline and concentration of reagents.

### 4.2 ∣ Peptide synthesis

Peptides were synthesized as described in previous work. Briefly, an automated Eclipse peptide synthesizer (AAPPTec) was utilized for standard solid-phase Fmoc synthesis on Rink-amide resin (0.55 mmol/g, 200 mg). Amino acids were coupled (C to N) under nitrogen protection with 0.2 M Fmoc-amino acid (5 equiv.) in 3 mL of dimethylformamide (DMF), 0.2M HBTU (5 equiv.) in 3 mL of DMF, 0.4 M DIPEA (7.5 equiv.) in 3 mL of DMF, and 20% (v/v) piperidine in 2 × 4 mL of DMF for each coupling cycle. The resulting resin and peptide were then transferred to a syringe filter (Torviq Inc.) and washed with three rounds of DMF (5 mL each) and three rounds of dichloromethane (DCM) (4 mL each). It was then dried under a vacuum. For the acetylated peptides, the N-terminus was acetylated using the following recipe: 4 mL of DMF, 0.5 mL of pyridine, and 0.5 mL of acetic anhydride. The solution was then subjected to light stirring for 30 min before being purged and washed with DMF and DCM as previously described. The dried peptides were subsequently cleaved from the resin using a 5 mL cocktail solution that consisted of 83% trifluoroacetic acid (TFA), 5% H2O, 5% thioanisole, 5% phenol, and 2% EDDET. The incubated solution was gently rotated for 2 h. The resin was then filtered, and the filtrate containing the crude peptide was collected and precipitated using cold ethyl ether (20 mL, −20°C) and centrifuged three times (8000 rpm, 3 min). Once the supernatant was removed, the precipitated pellets were dried and resuspended in 10 mL of acetonitrile (ACN)/H_2_O mixtures, and the percentage of ACN was controlled based on the solubility of the peptide.

### 4.3 ∣ Peptide purification and characterization

Peptide purification was conducted as described by Amer et al.^[31]^ Once dry, the crude peptides were purified with a Shimadzu LC-40 HPLC system equipped with an LC-40D solvent delivery module, SPD-M40 photodiode array detector, and DGU-403 degassing unit. Two milliliters was injected with a Zorbax 300 BS C18 column (5 μM, 9.4 × 250 mm) using an elution flow rate of 5 mL/min over a 40-minute gradient from 10% to 95% acetonitrile in water (0.05% TFA). The peptide bond absorbance at 220 nm was monitored closely, and the eluate was collected for characterization. ESI-MS in positive ion mode via a Micromass Quattro Ultima mass spectrometer provided by the Molecular MS Facility (MMSF) at UC San Diego was utilized with a MEOH/H2O mixture (1:1, v/v) and an injection volume of 5 μL. Some compounds were characterized using a matrix-assisted laser desorption/ionization-time of flight (MALDI-TOF) mass spectrometer in linear positive mode. Here, a-cyano-4-hydroxycinnamic acid (HCCA) was used as the matrix at a ratio of 1:3, and 2 μL was placed and dried with a heat gun for analysis. Duplicates were used as recommended by the MMSF. Fractions containing the pure peptide as confirmed by ESI-MS (positive ion mode) were lyophilized in a FreeZone Plus 2.5 freeze-dry system (Labconco Corp.) and aliquoted and stored under dry conditions at 2°C for further use.

### 4.4 ∣ Peptide-particle incubation

The optical properties of the particles were analyzed as follows: 100 μL of BSPP-AgNPs was gently mixed with different concentrations of peptides in a 96-well plate. After 30 min, the UV–vis spectra were recorded using a hybrid multimode microplate reader (Synergy H1 model, BioTek Instruments, Inc.).

### 4.5 ∣ Transmission electron microscopy

TEM images were taken using a JEOL 1200 EX II instrument operated at 80 kV. The TEM grids were prepared by drop casting 2 μL of each sample followed by air drying overnight. Diffraction patterns were collected using a ThermoFisher Talos 200X G2 S/TEM instrument operated at 200 keV.

### 4.6 ∣ Scanning electron microscopy

A Phenom G6 Pro SEM from NanoScience Instruments (Thermo Fisher) was used with a cesium hexaboride source. Mixed back-scattered detection and secondary electron detection were used. The SEM stage was prepared by drop casting 2 μL of each sample followed by air drying overnight. The samples were completely dried before imaging.

### 4.7 ∣ Image processing

Image processing was conducted using ImageJ v5. The FracLac extension was used for digital image analysis.

### 4.8 ∣ Bradford assay

Note that, 100 μL of each standard or unknown sample was pipetted into a microplate well. Next, 100 μL of the Bradford reagent was added to each well and mixed with a plate shaker for 30 seconds. The plate was then incubated for 5 min at room temperature and the absorbance was measured 595 nm on a plate reader. The average 595 nm measurement for the blank replicates was subtracted from the 595 nm measurements of all other standard and unknown sample replicates.

## Supplementary Material

si

## Figures and Tables

**TABLE 1:** Diffusion coefficients.

Varied parameter	Value	Diffusion coefficient $\left(d_{H}\right)$
Diameter, D	10 nm	6.28 × 10^−11^ m^2^/s
	20 nm	3.14 × 10^−11^ m^2^/s
	60 nm	1.05 × 10^−11^ m^2^/s
Viscosity, η	11.9 × 10^−4^ Pa·s	1.45 × 10^−13^ m^2^/s
Temperature, T	37°C	3.29 × 10^−11^ m^2^/s
	8°C	2.98 × 10^−11^ m^2^/s

**TABLE 2:** Peptides.

	Sequence	Label	Charge	Objective
p0	TSG	TSG	0	No net charge
p1	RRK	RRK	+3	Electrostatic interactions
p2	RRKRRK	${\left(\text{RRK}\right)}_{2}$	+6	Effect of repeating the bridging motif (n=2,3)
p3	RRKRRKRRK	${\left(\text{RRK}\right)}_{3}$	+9	
p4	KKK	KKK	+3	Electrostatic interactions
p5	KKKKKK	${\left(\text{KKK}\right)}_{2}$	+6	Effect of repeating the bridging motif (n=2,3)
p6	KKKKKKKKK	${\left(\text{KKK}\right)}_{3}$	+9	
p7	HHK	HHK	+1	Electrostatic/ π-π interactions
p8	HHKHHK	${\left(\text{HHK}\right)}_{2}$	+2	Effect of repeating the bridging motif (n=2,3)
p9	HHKHHKHHK	${\left(\text{HHK}\right)}_{3}$	+3	
p10	FFK	FFK	+1	π-π/ hydrophobic interactions
p11	FFKFFK	${\left(\text{FFK}\right)}_{2}$	+2	Effect of repeating the bridging motif (n=2,3)
p12	FFKFFKFFK	${\left(\text{FFK}\right)}_{3}$	+3	
p13	WWK	WWK	+1	π-π/ hydrophobic interactions
p14	WWKWWK	${\left(\text{WWK}\right)}_{2}$	+2	Effect of repeating the bridging motif (n=2,3)
p15	WWKWWKWWK	${\left(\text{WWK}\right)}_{3}$	+3	
p16	RTSGR	RTSGR	+2	Non-bridging spacer
p17	RTSGTSGR	$R{\left(\text{TSG}\right)}_{2}R$	+2	Effect of repeating the non-bridging spacer (n=2,3)
p18	RTSGTSGTSGR	$R{\left(\text{TSG}\right)}_{3}R$	+2	
p19	RRTSGRR	RRTSGRR	+4	Increased charge density
p20	RRTSGTSGRR	$\text{RR}{\left(\text{TSG}\right)}_{2}\text{RR}$	+4	Effect of repeating the non-bridging spacer (n=2,3)
p21	RRTSGTSGTSGRR	$\text{RR}{\left(\text{TSG}\right)}_{3}\text{RR}$	+4	
