# Supplementary material for: Mechanism of hierarchical plasmonic biomaterials engineered through peptide-directed self-assembly
Source: Aggregate (Hoboken). Author manuscript; Available in PMC 2025 Sep 17. (PMC12439837; doi:10.1002/agt2.677)
Supplement: si [file NIHMS2071469-supplement-si.pdf]

SUPPLEMENTAL INFORMATION

Mechanism of hierarchical plasmonic biomaterials engineered through peptide-directed self-assembly.

Lubna Amer,[a] Maurice Retout,[b] Zhicheng Jin,[b] Sumathi Kakanar, [b] and Jesse V. Jokerst[a,b,c*]

[a] Program in Materials Science and Engineering, University of California, San Diego, La Jolla, CA 92093, United States

[b] Aiiso Yufeng Li Family Department of Chemical and Nano Engineering, University of California, San Diego, La Jolla, CA 92093, United States

[c] Department of Radiology, University of California, San Diego, La Jolla, CA 92093, United States

***Corresponding author's email:** jjokerst@ucsd.edu (J.V.J.)

Table of Contents

I. Materials ____ **2**

II. Instrumentation ____ **2**

III. Supplementary Data ____ **4**

BSPP-AgNPs Characterization ____ *4*

Peptide Characterization ____ *7*

Assembly Characterization ____ *10*

IV. Author Contributions ____ **38**

I. Materials

Fmoc-protected L-amino acids, hexafluorophosphate benzotriazole tetramethyl uranium (HBTU), and Fmoc-Rink amide MBHA resin (0.67 mmol/g, 100-150 mesh) were purchased from AAPPTec, LLC (Louisville, KY). Organic solvents including N,N-dimethylformamide (DMF, sequencing grade), acetonitrile (ACN, HPLC grade), ethyl ether (certified ACS), methylene chloride (DCM, certified ACS), and dimethyl sulfoxide (DMSO, certified ACS) were from Fisher Scientific International, Inc. (Hampton, NH). Thioanisole (>99%), N,N-diisopropylethylamine (DIPEA, >99%), and triisopropylsilane (TIPS, >98%) were purchased from Tokyo Chemical Industry Co., Ltd. (TCI). Ultrapure water (18 MΩ.cm) was obtained from a Milli-Q Academic water purification system (Millipore Corp., Billerica, MA).

Bis(p-sulfonatophenyl)phenylphosphine dihydrate dipotassium salt (BSPP, 97%), gold(III) chloride trihydrate (HAuCl4 .3H2O, >99.9%), sodium citrate tribasic dihydrate (>99%), Trizma® base (>99.9%), Trizma® hydrochloride (>90%), trifluoroacetic acid (TFA, HPLC grade, >99%), 2,2′-(ethylenedioxy)diethanethiol (EDDET, 95%), piperidine (ReagentPlus® , 99%) and Neuraminidase (NA) from Clostridium perfringens were purchased from Sigma Aldrich (St. Louis, MO). Sodium chloride (certified ACS), sodium phosphate monobasic monohydrate (certified ACS), and sodium phosphate dibasic anhydrous (certified ACS) were purchased from Fisher Scientific International, Inc. (Hampton, NH). Bradford Protein Assay with BSA Protein Standard, 2500 Microplate Assays/250 Test Tube Assays was purchased from Steller Scientific.

TEM grids (formvar/carbon 300 mesh Cu) were purchased from Ted Pella (Redding, CA). Amicon® ultra-15 centrifugal filter units (M.W. cutoff =100 kDa) and automation compatible syringe filters (PTFE, 0.45 mm) were from MilliporeSigma (St. Louis, MO). Glassware and stir bars were cleaned with aqua regia (HCl:HNO3=3:1 by volume) and boiling water before use. Some peptides were purchased from Genscript Inc. (New Jersey, USA).

II. Instrumentation

The optical absorption measurements were collected using a hybrid multi-mode microplate reader (Synergy™ H1 model, BioTek Instruments, Inc.) in a 96-well plate. Peptides were synthesized using an automated Eclipse™ peptide synthesizer (AAPPTec, Louisville, KY) through standard solid phase Fmoc syntheses on Rink-amide resin. Peptides were lyophilized in a FreeZone Plus 2.5 freeze dry system (Labconco Corp., Kansas, MO). Peptide purification was carried out using a Shimadzu LC-40 HPLC system equipped with a LC-40D solvent delivery module, photodiode array detector SPD-M40, and degassing unit DGU-403. The sample was dissolved in water and acetonitrile and applied on a Zorbax 300 BS, C18 column (5 mm, 9.4×250 mm) from Agilent, and eluted at 1.5 mL/min with a 40 min gradient from 10% to 95% solvent B. Here, solvent A is water (0.05% TFA), and solvent B is acetonitrile (0.05% TFA). Preparative injections were monitored at 190, 220, and 254 nm. All products were purified by HPLC to reach purity of >95%.

Electrospray ionization mass spectrometry (ESI-MS) data was acquired by using a Micromass Quattro Ultima mass spectrometer in the Molecular MS Facility (MMSF) at Chemistry and Biochemistry Department, UC San Diego. ESI-MS samples were prepared in a 50% MeOH/H2O mixture. Peptide concentration was determined using a NanoDrop™ One UV-vis spectrophotometer (Thermo Fisher Scientific, Waltham, MA).

A Malvern Instruments™ Zetasizer Nano™ was used to determine the nanoparticle size and charge from a single sample filled into a small cuvette. This instrument features a multipurpose titrator, which allows measurement of the average zeta potential (surface charge) of the

nanoparticles. A Nicolet™ iS50 Fourier transform infrared (FTIR) spectrometer, fitted with Smart-iTR™ diamond Attenuated Total Reflectance (ATR) attachment for ATR measurements was used to characterize the chemical composition of nanoparticles. The multi-laser nanoparticle tracking analysis (M-NTA) data was collected with the ViewSizer 3000 (Horiba scientific, CA, USA). Automated noise analysis determines the optimal wavelength for representing each nanoparticle. Here, 8-bit composite videos were generated, and 10 videos were used per analysis (300 frames for seconds). A quartz cuvette filled with 1 mL of each sample was used for the measurement. The operating temperature was set up to 22 °C.

III. Supplementary Data

<u>BSPP-AgNPs Characterization</u>

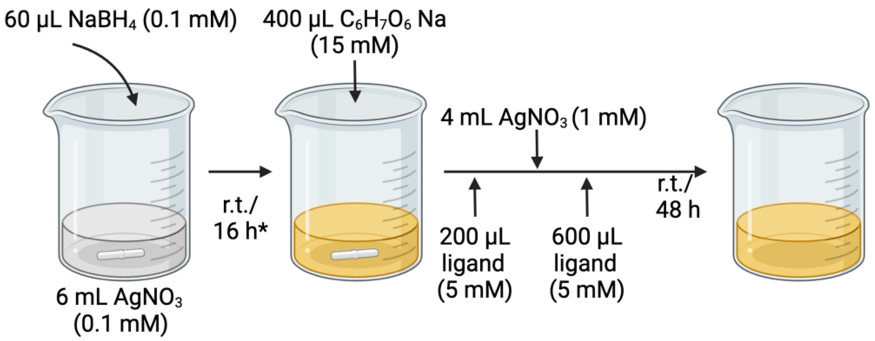

Figure S1. BSPP-AgNPs two-step seed growth procedure.

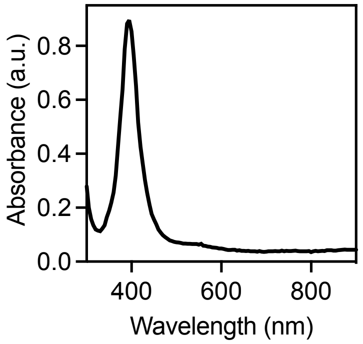

Figure S2. BSPP-AgNPs UV-visible spectrum confirm the synthesis as defined by the peak at 400 nm, representative of 20 nm silver nanoparticles.

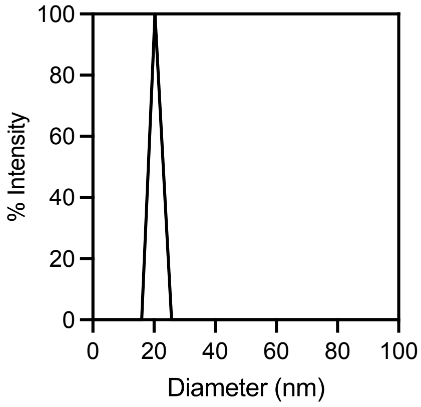

Figure S3. BSPP-AgNPs DLS illustrate the homogeneity of the particle size at 20 nm. PDI = 0.124.

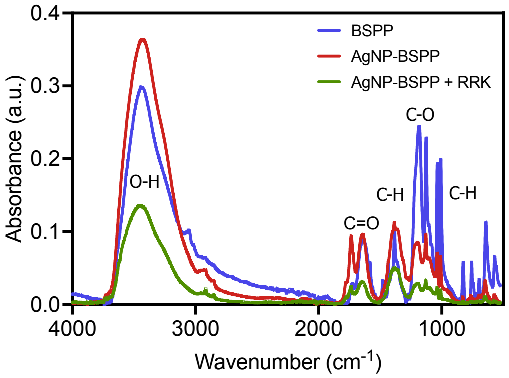

Figure S4. BSPP-AgNPs FT-IR spectra BSPP has been successfully coated on the silver nanoparticles. The S-O bands reflect the presence of the (bis(p-sulfonatophenyl)phenylphosphine) (BSPP) ligand. To prove that we first studied the BSPP ligand alone (blue curve). The peaks at 1180 cm^{-1} and 1010 cm^{-1} can be attributed to the S-O bonds in BSPP due to the characteristic vibrational modes of the sulfonate groups ($-SO_3^-$). BSPP-coated nanoparticles (red), confirms coating of the BSPP ligand on the silver nanoparticle surface. After nanoparticle assembly by the RRK peptide (green), we observe subtle shifts in relative intensities, indicating alterations in the vibrational modes of specific functional groups. The intensity of the S-O bands decreases suggesting loss of ligand. However, no new bands suggestive of covalent bond formation appear, which suggests that the interactions between the peptide and the nanoparticle is primarily driven by non-covalent interactions rather than chemical modification of the peptide functional groups.

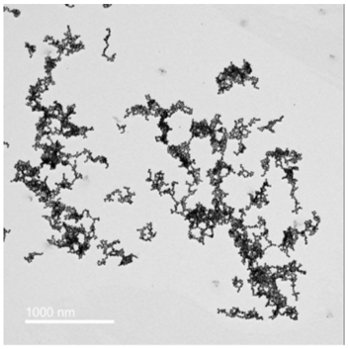

Figure S5. BSPP-AgNPs TEM show individually dispersed nanoparticles of similar size and a lack of coalescence.

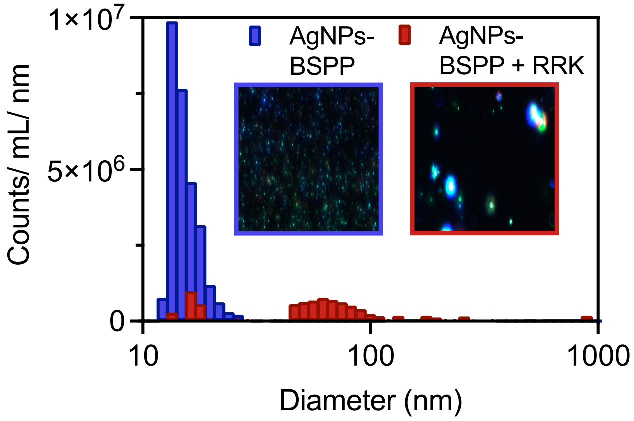

Figure S6. Dynamic evolution of the assembly of BSPP-AgNPs. M-NTA spectra before (blue) and after (red) the addition of 1 μM RRK confirm that RRK induces nanoparticle assembly. Small nanoparticles (<200 nm) scatter in blue while large nanoparticles (>300 nm) scatter in green and red.

Peptide Characterization

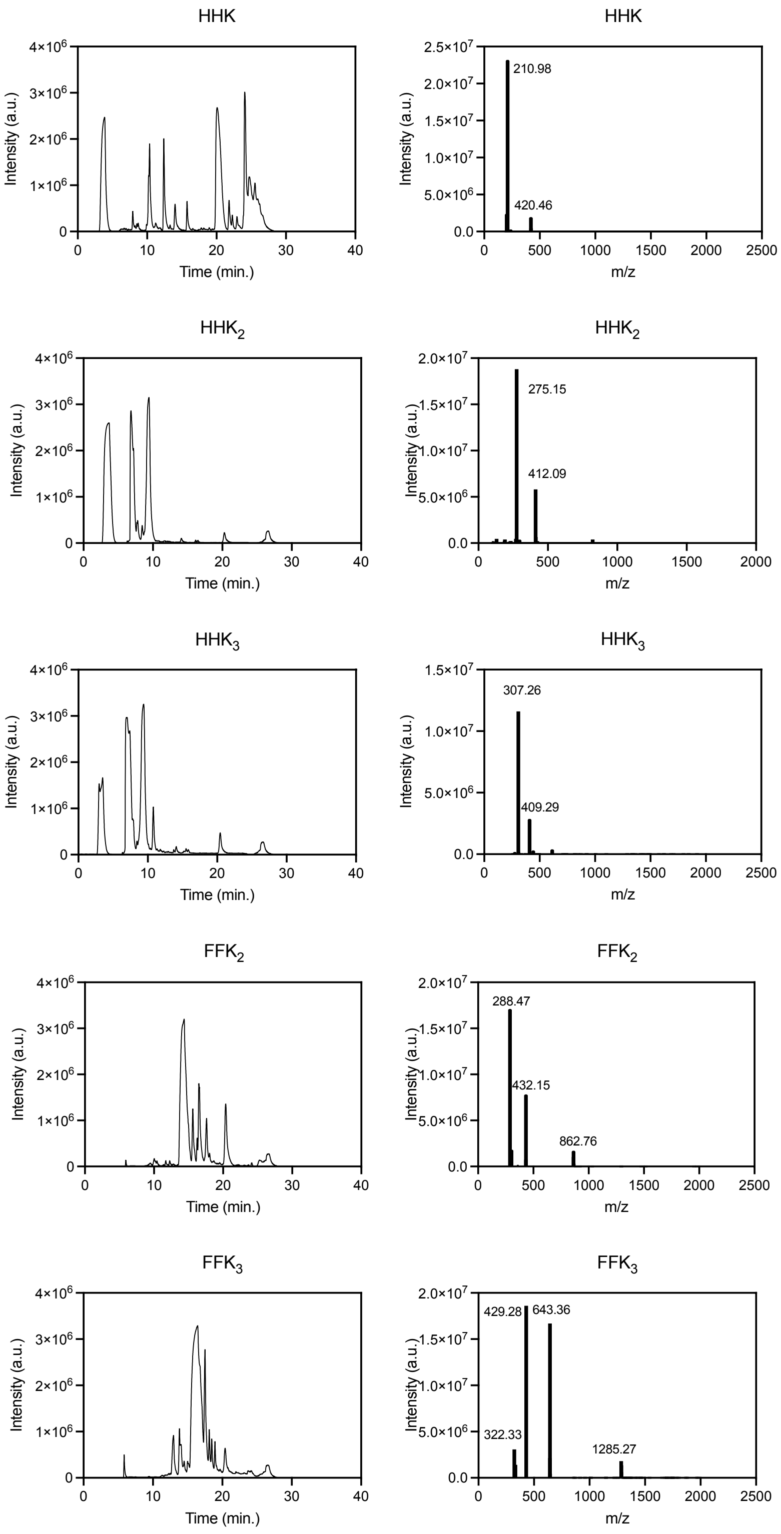

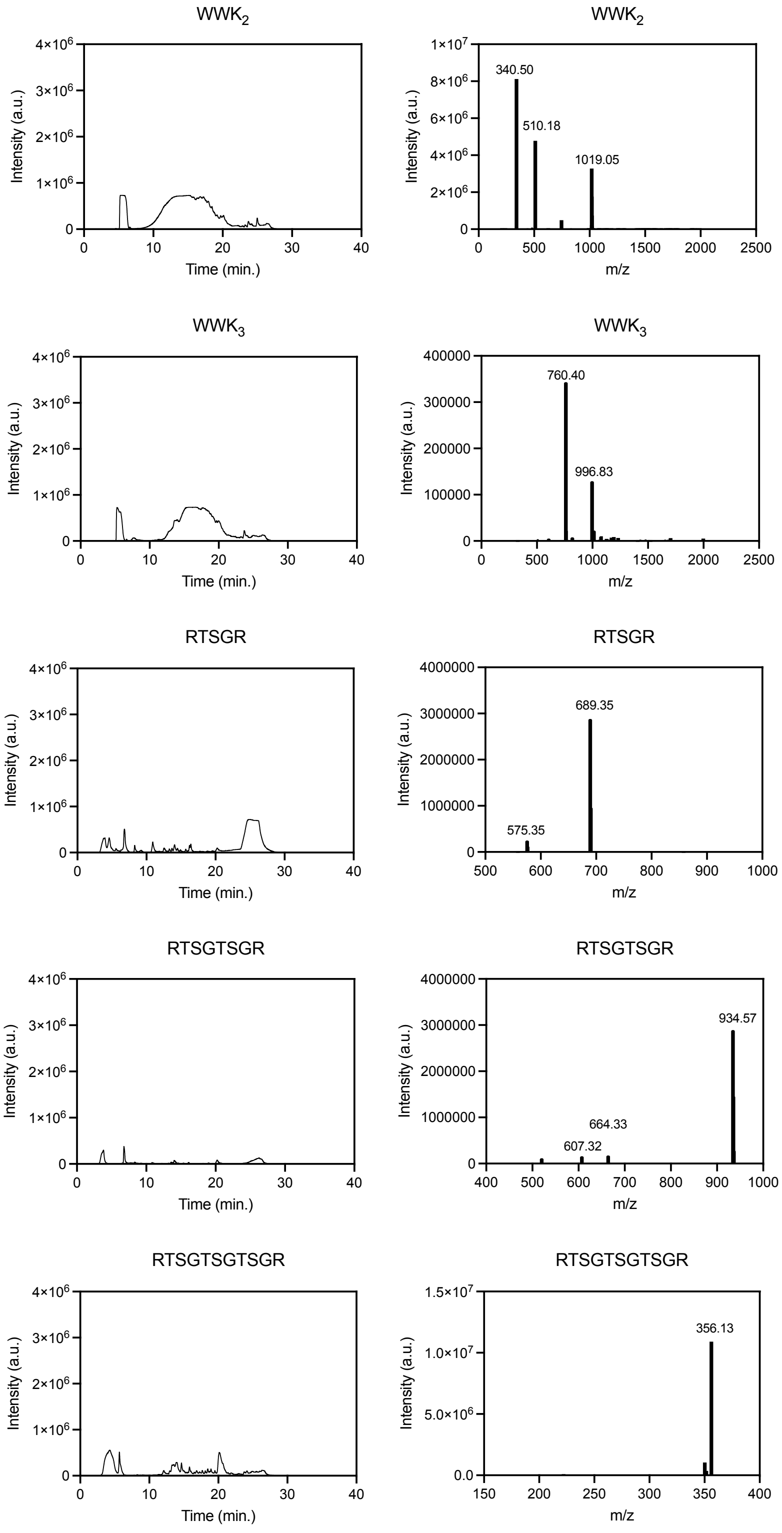
WWK$_2$
Intensity (a.u.)
Time (min.)
WWK$_2$
340.50
510.18
1019.05
Intensity (a.u.)
m/z
WWK$_3$
Intensity (a.u.)
Time (min.)
WWK$_3$
760.40
996.83
Intensity (a.u.)
m/z
RTSGR
Intensity (a.u.)
Time (min.)
RTSGR
689.35
575.35
Intensity (a.u.)
m/z
RTSGTSGR
Intensity (a.u.)
Time (min.)
RTSGTSGR
934.57
664.33
607.32
Intensity (a.u.)
m/z
RTSGTSGTSGR
Intensity (a.u.)
Time (min.)
RTSGTSGTSGR
356.13
Intensity (a.u.)
m/z

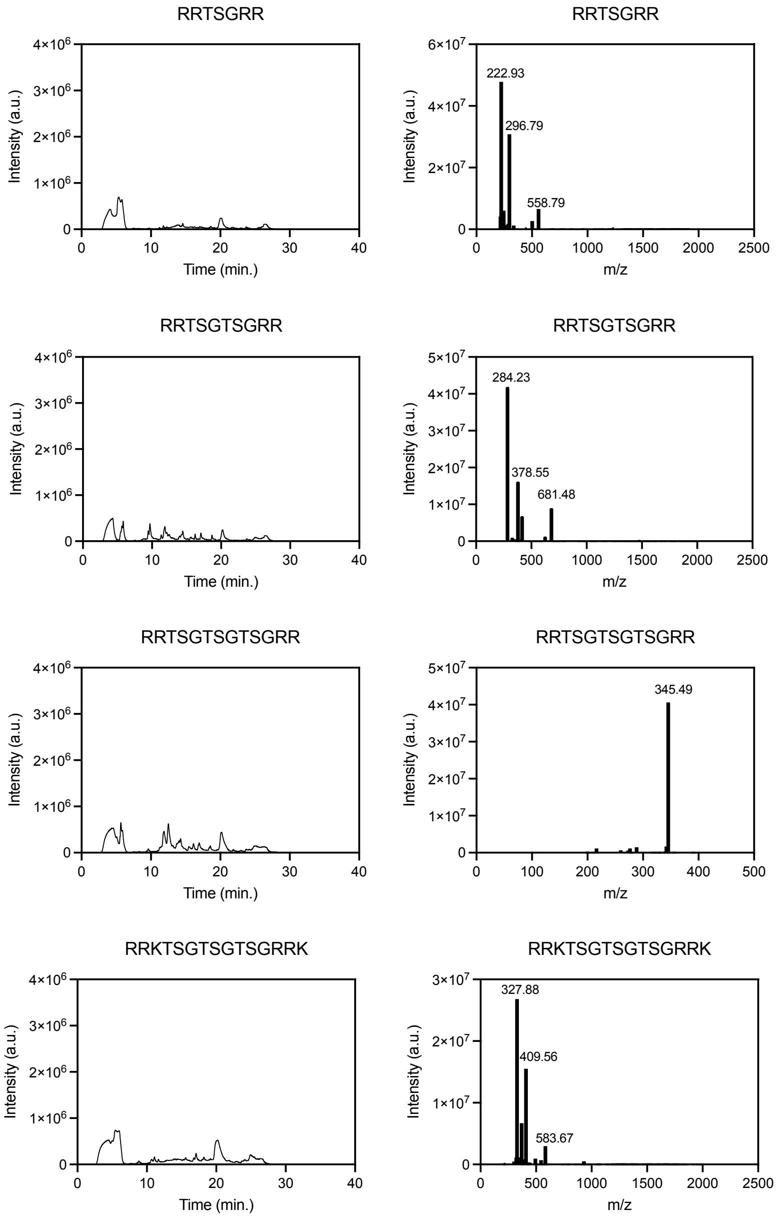

Figure S7. HPLC and ESI-MS data of the synthesized peptides. HPLC is shown in the left panel, and MS of purified fraction in the right panel showing high purity content for all synthesized peptides.

Assembly Characterization

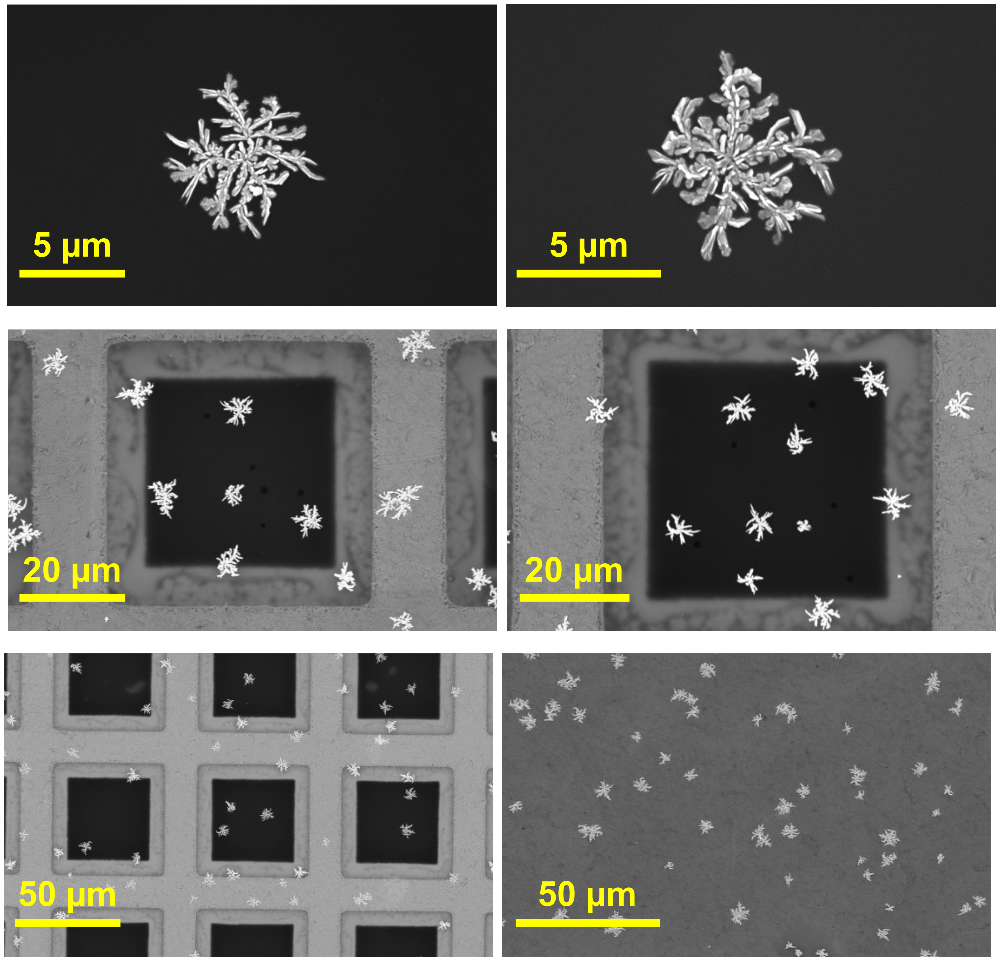

Figure S8. SEM images of BSPP-AgNPs after 30-minute incubation at room temperature with 1 µM RRK at multiple magnifications showing fractal structures, complete coalescence, and absence of residual (free) nanoparticles.

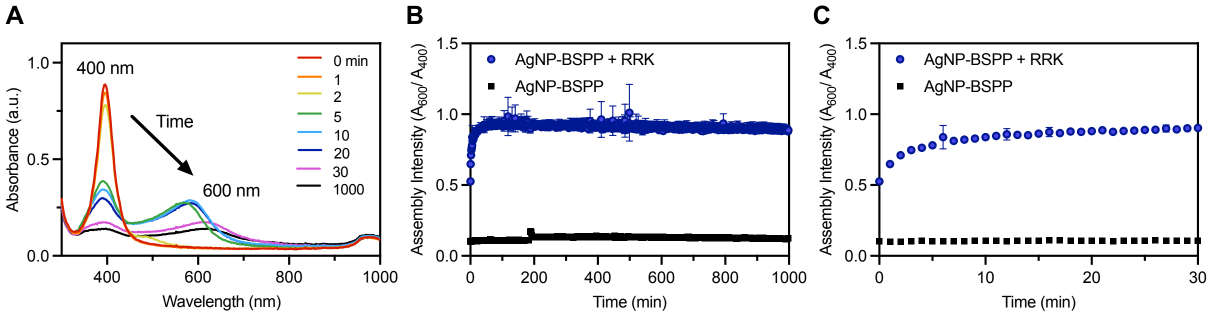

Figure S9. Evolution of the nanoparticle assembly over time. **(A)** UV-visible spectra of BSPP-AgNPs incubated with 1 µM RRK over time. **(B)** Kinetic spectra of AgNP assembly intensity (defined as Abs.600 nm/Abs.400 nm) over ~ 17 hours and **(C)** over 30 minutes where the plateau is first observed. The error bars represent standard deviation (n = 4).

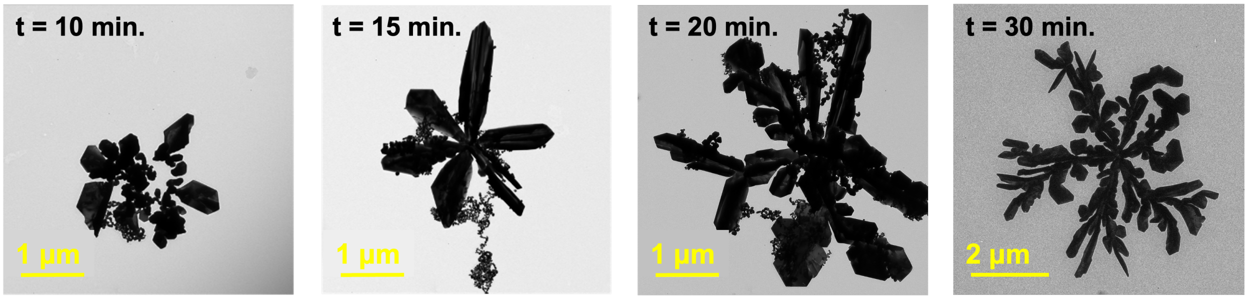

Figure S10. Time-dependent TEM images showing the evolution of the fractal assembly of the BSPP-AgNPs upon the addition of 1 µM RRK.

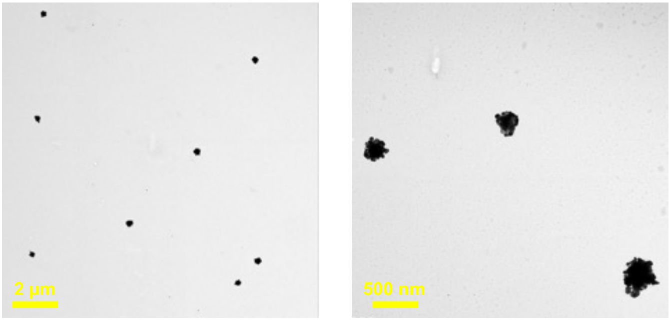

Figure S11. TEM image after 254 nm light exposure (7.22 mW/cm²) for 20 minutes.

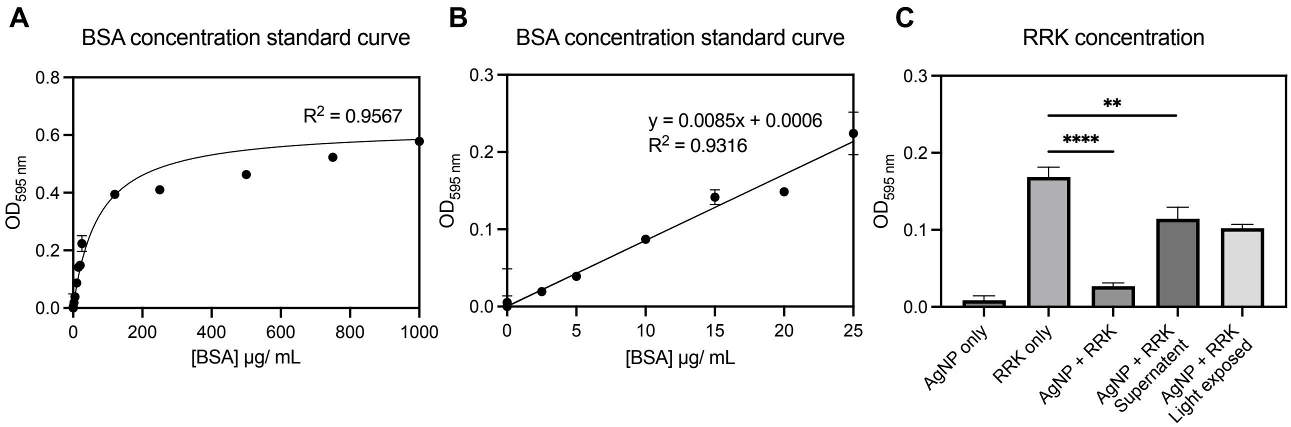

Figure S12. Quantification of proteins via the Bradford method. **(A)** Standard curve of BSA concentration from 0 – 2000 µg/ mL versus absorbance at 595 nm. The standard curve was prepared by plotting the average blank corrected 595 nm measurement for each BSA standard vs. its concentration in µg/mL. **(B)** Standard curve of BSA concentration from 0 – 25 µg/ mL versus absorbance at 595 nm. **(C)** Samples contained a decrease in RRK compared to starting concentration of peptide. $[RRK]_{only}$ = 19.78 µg/ mL; $[RRK]_{Fractal\ structures}$ = 3.076 µg/ mL; $[RRK]_{Fractal\ structure\ supernatant}$ = 11.94 µg/ mL; $[RRK]_{Fractal\ structures\ after\ light\ exposure}$ = 13.39 µg/ mL. Error bars represent the standard error for the mean for n = 4. Asterisks denote values from a two-tailed t-test (****p < 0.00001; **p < 0.001). Note: A 10x concentration of RRK was used to accommodate the window of the assay.

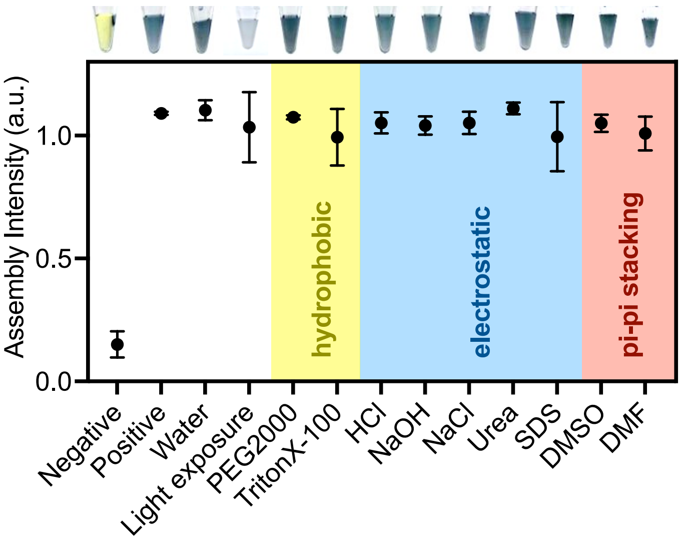

Figure S13. Quantified reversal color change of the peptide/BSPP-AgNP aggregates in different surfactant solutions (10 mM, 100 µL) or solvents (100 µL). The negative control is BSPP-AgNPs only, and positive control is the peptide/BSPP-AgNPs aggregates only. The yellow area indicates dominant hydrophobic interactions, the blue area indicates prevalent electrostatic interactions, and the red area implies strong π–π stacking forces. Error bars = standard deviations (n = 3). This shows that the particles cannot be successfully redispersed because they have coalesced to form new structures.

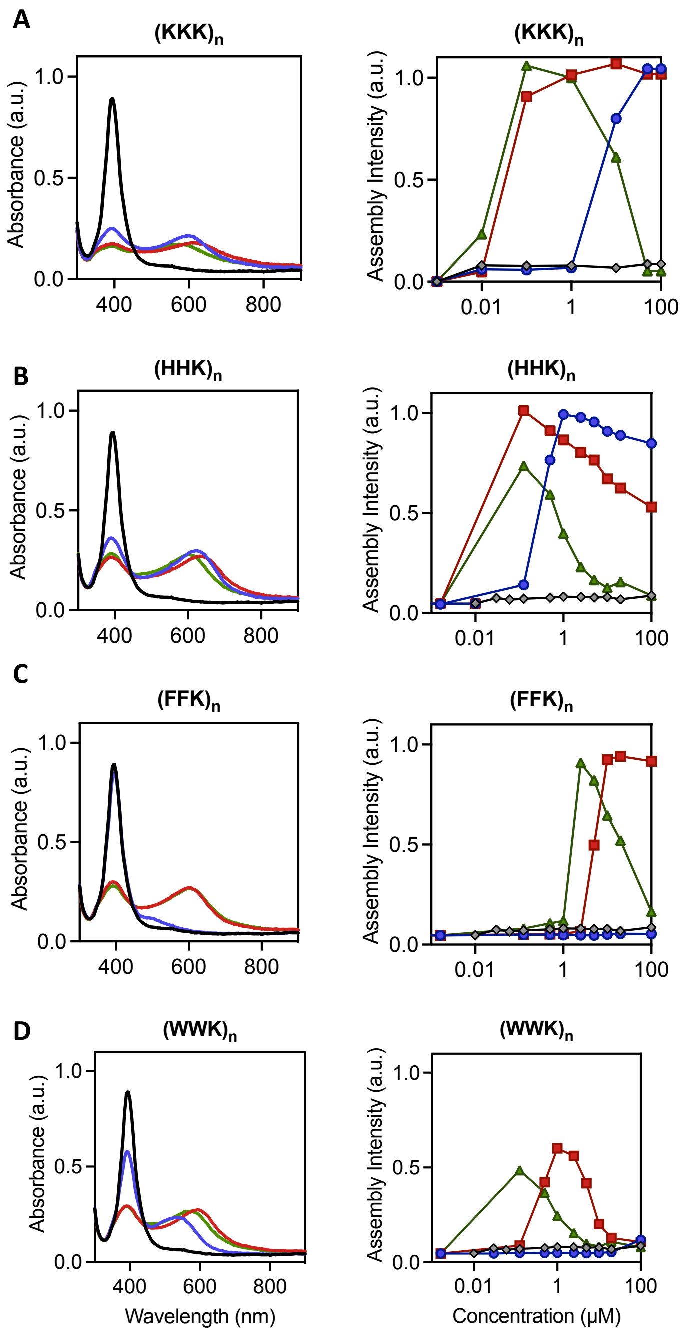

Figure S14. UV-Vis spectra as a function of absorbance (left) and peak absorbance assembly intensity (Abs. 600/ Abs. 400 nm) (right) of several samples of BSPP-AgNPs in the presence of different concentrations of **(A)** Lysine-based peptide family. **(B)** Histidine-based peptide family. **(C)** Phenylalanine-based peptide family. **(D)** Tryptophan-based peptide family. Black curve: TSG; blue curve: XXK; green curve: $(XXK)_2$; red curve: $(XXK)_3$. This shows that an increase in the number of repetitions of the bridging motif resulted in an earlier redshift at lower peptide concentrations: $(XXK)_3$ > $(XXK)_2$ > XXK.

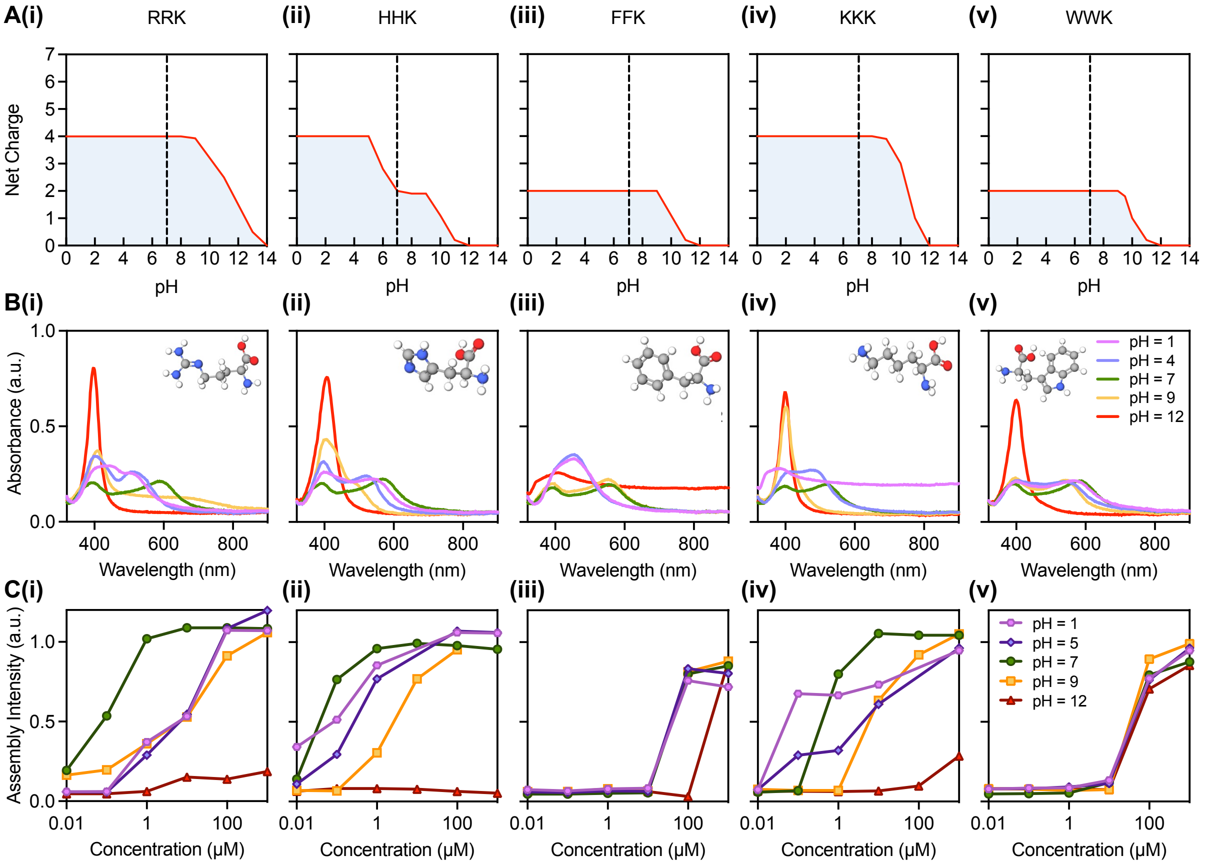

Figure S15. pH dependent interactions of peptide-induced assembly. **(A)** Isoelectric point of synthesized peptides. (i) Arginine-based peptide (RRK). (ii) Histidine-based peptide (HHK). (iii) Phenylalanine-based peptide (FFK). (iv) Lysine-based peptide (KKK). (v) Tryptophan-based peptide (WWK). **(B)** UV—vis spectra along increasing pH. The shift towards the 400 nm peak demonstrates a pH dependence of the system likely due to the neutralization of both the BSPP ligand and the charged peptides. **(C)** Concentration titration of the monomeric peptides showing that RRK, HHK and KKK are most effected by the change in pH whereas FFK and WWK show little change. This highlights the electrostatic peptides and confirms the responsibility of other noncovalent interactions for FFK and WWK.

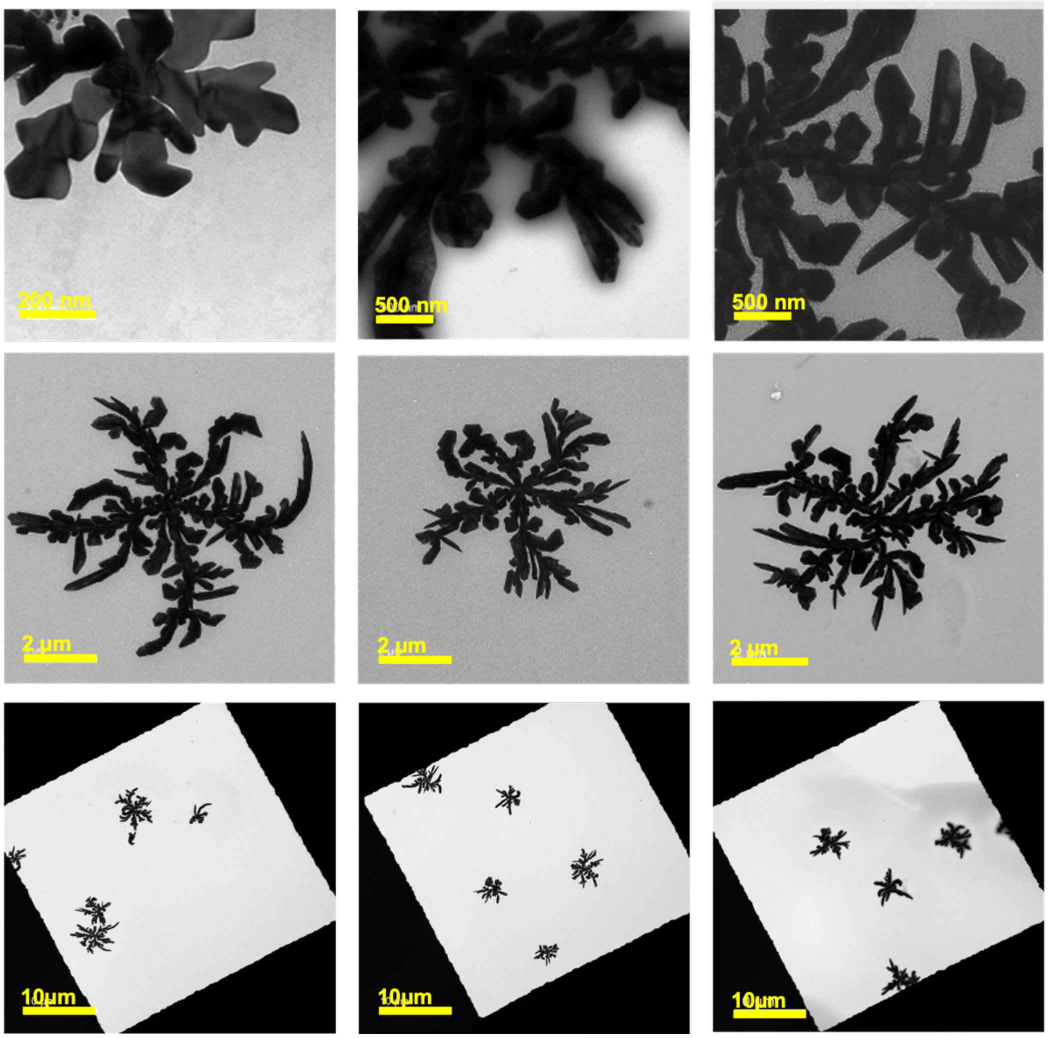

Figure S16. TEM images of BSPP-AgNPs after the addition of 1 μM of RRK at multiple magnifications showing fractal structures, complete coalescence, and absence of residual (free) nanoparticles.

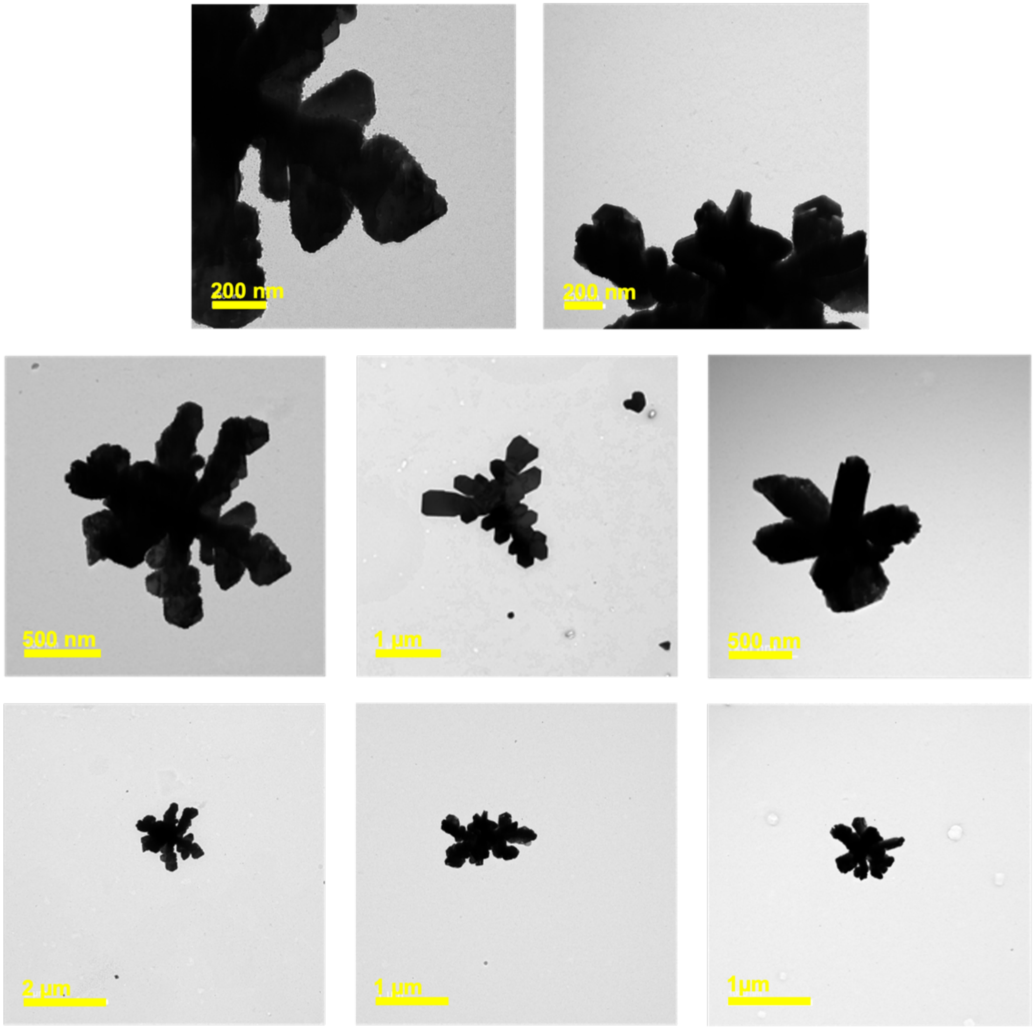

Figure S17. TEM images of BSPP-AgNPs after the addition of 1 µM of $(RRK)_2$ at multiple magnifications showing dense fractal structures with close proximity between the fractal branches.

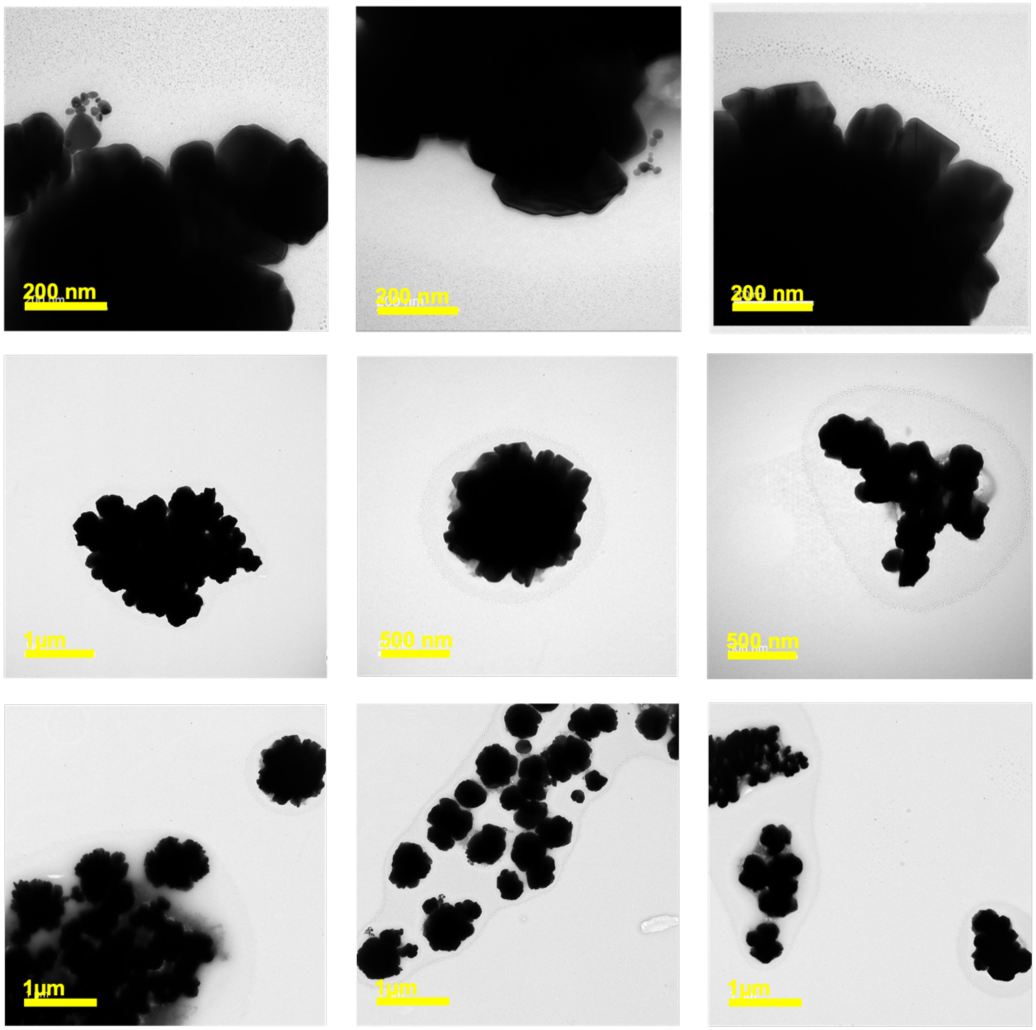

Figure S18. TEM images of BSPP-AgNPs after the addition of 1 μM of $(RRK)_3$ at multiple magnifications showing highly dense structures.

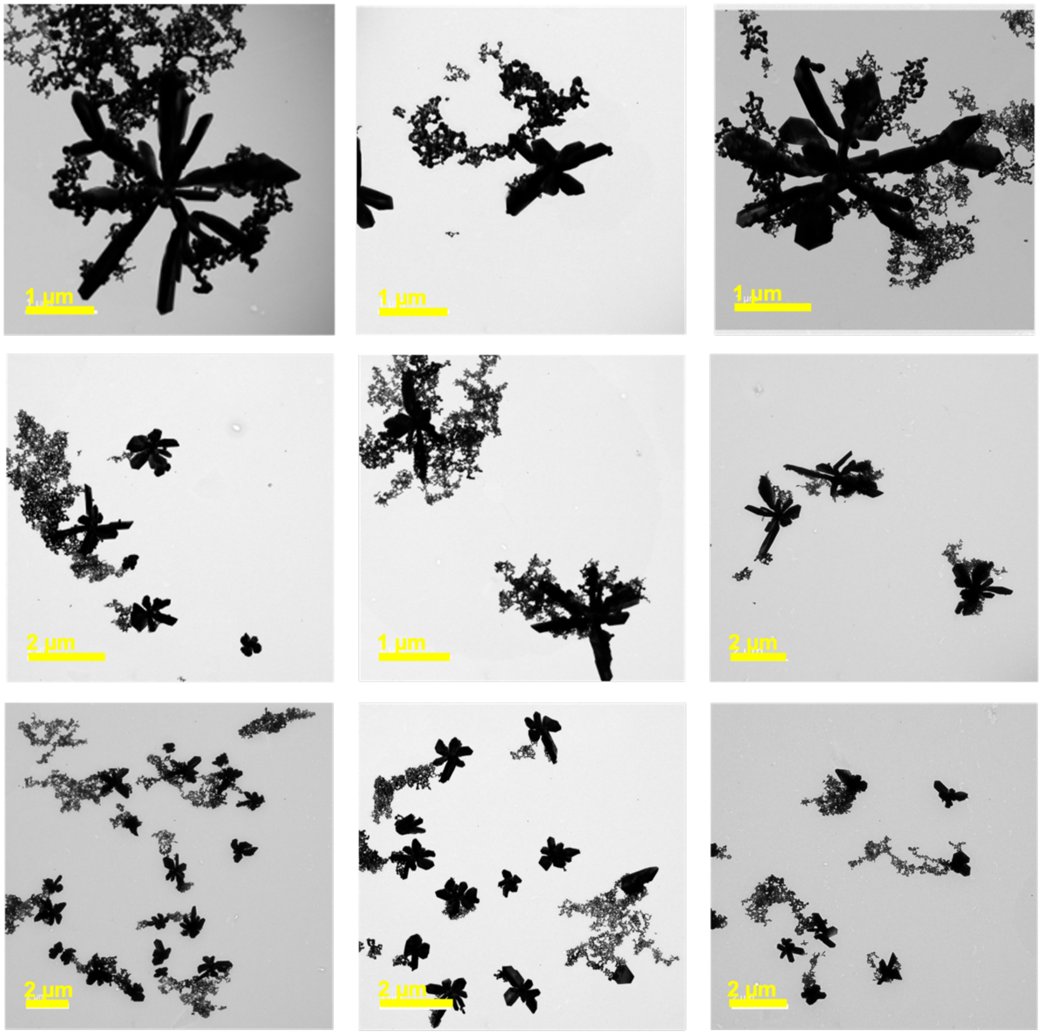

Figure S19. TEM images of BSPP-AgNPs after the addition of FFK at multiple magnifications.

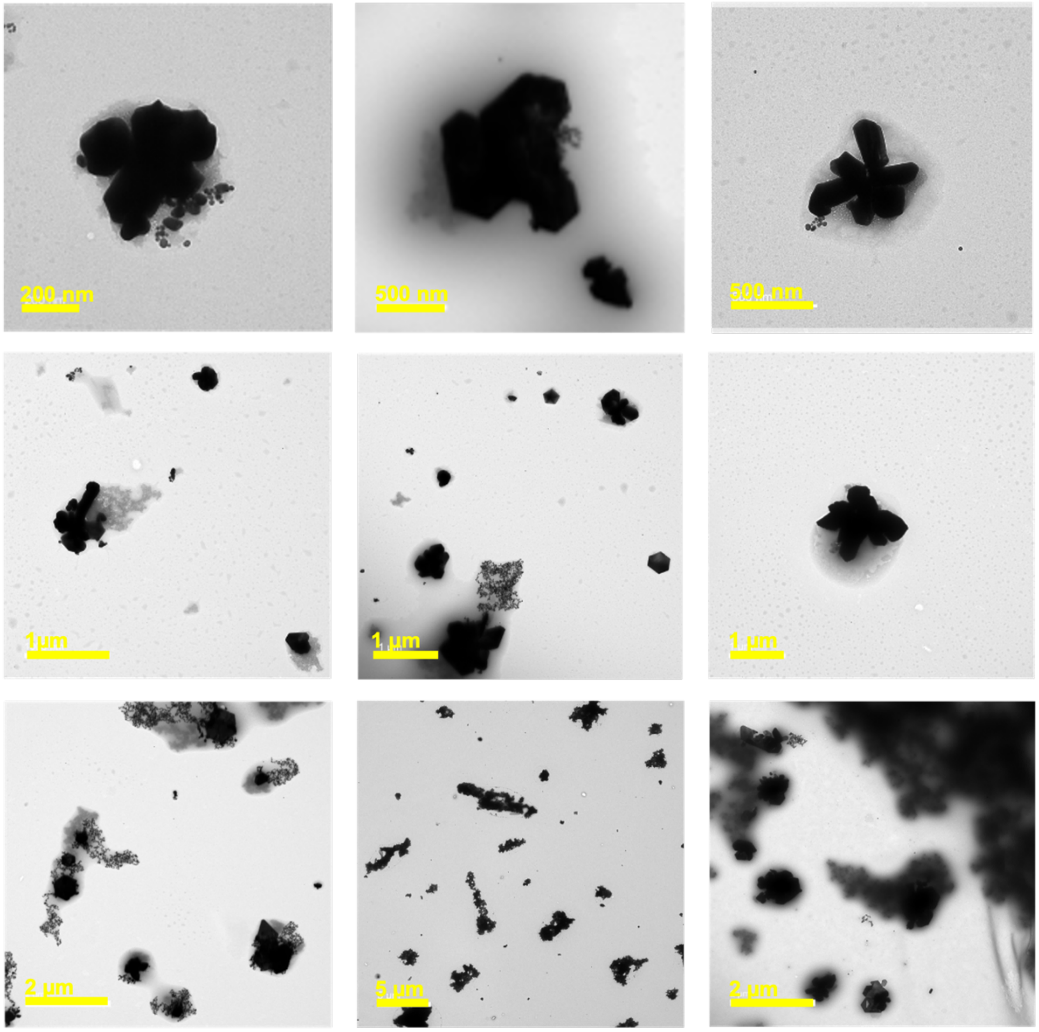

Figure S20. TEM images of BSPP-AgNPs after the addition of $(FFK)_2$ at multiple magnifications.

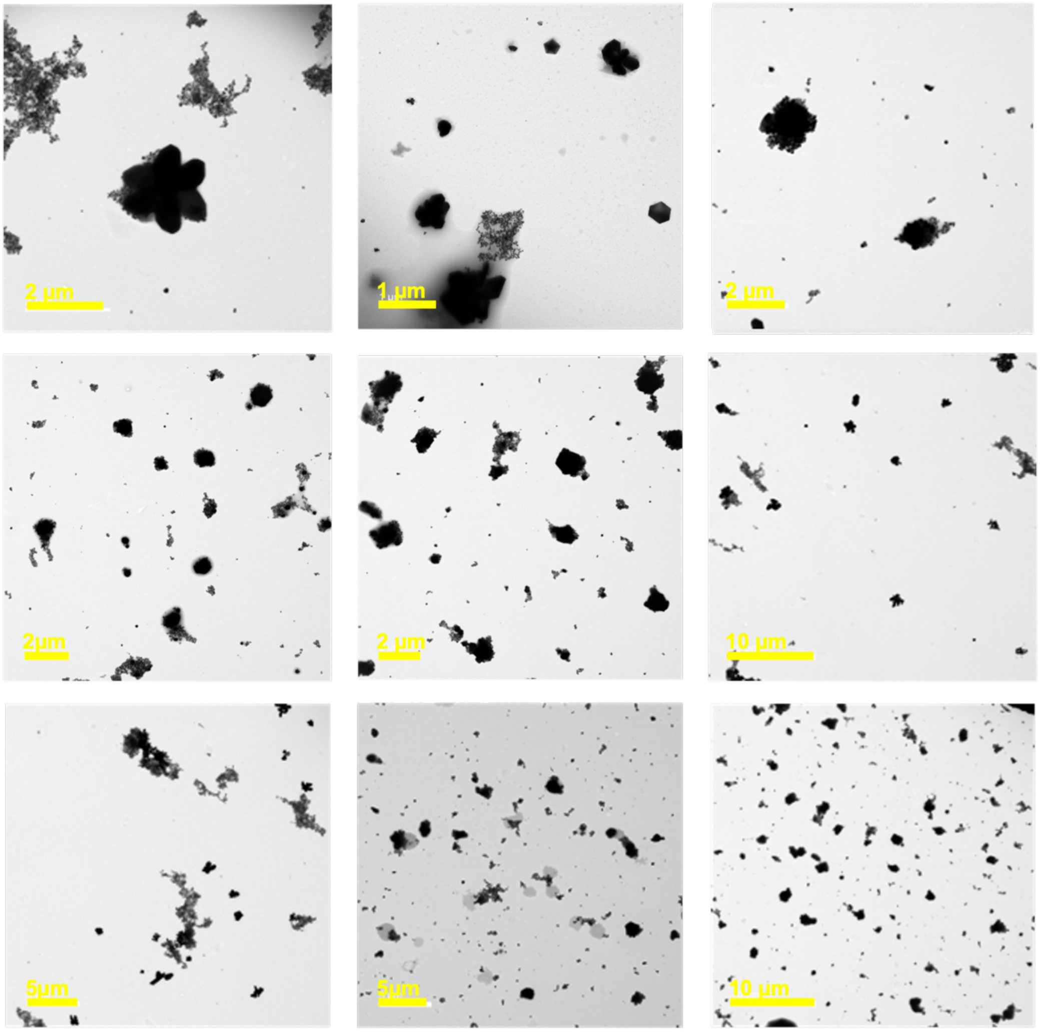

Figure S21. TEM images of BSPP-AgNPs after the addition of $(FFK)_3$ at multiple magnifications.

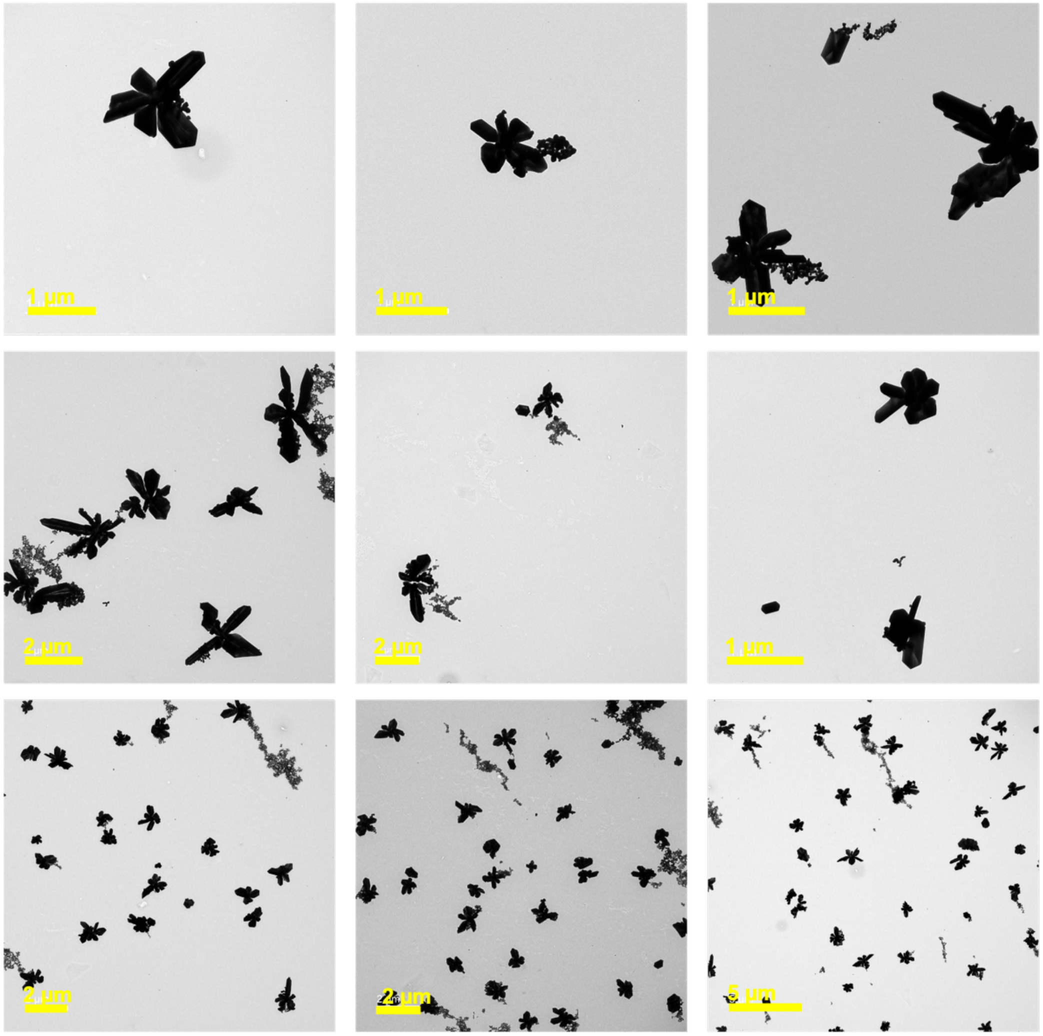

Figure S22. TEM images of BSPP-AgNPs after the addition of KKK at multiple magnifications.

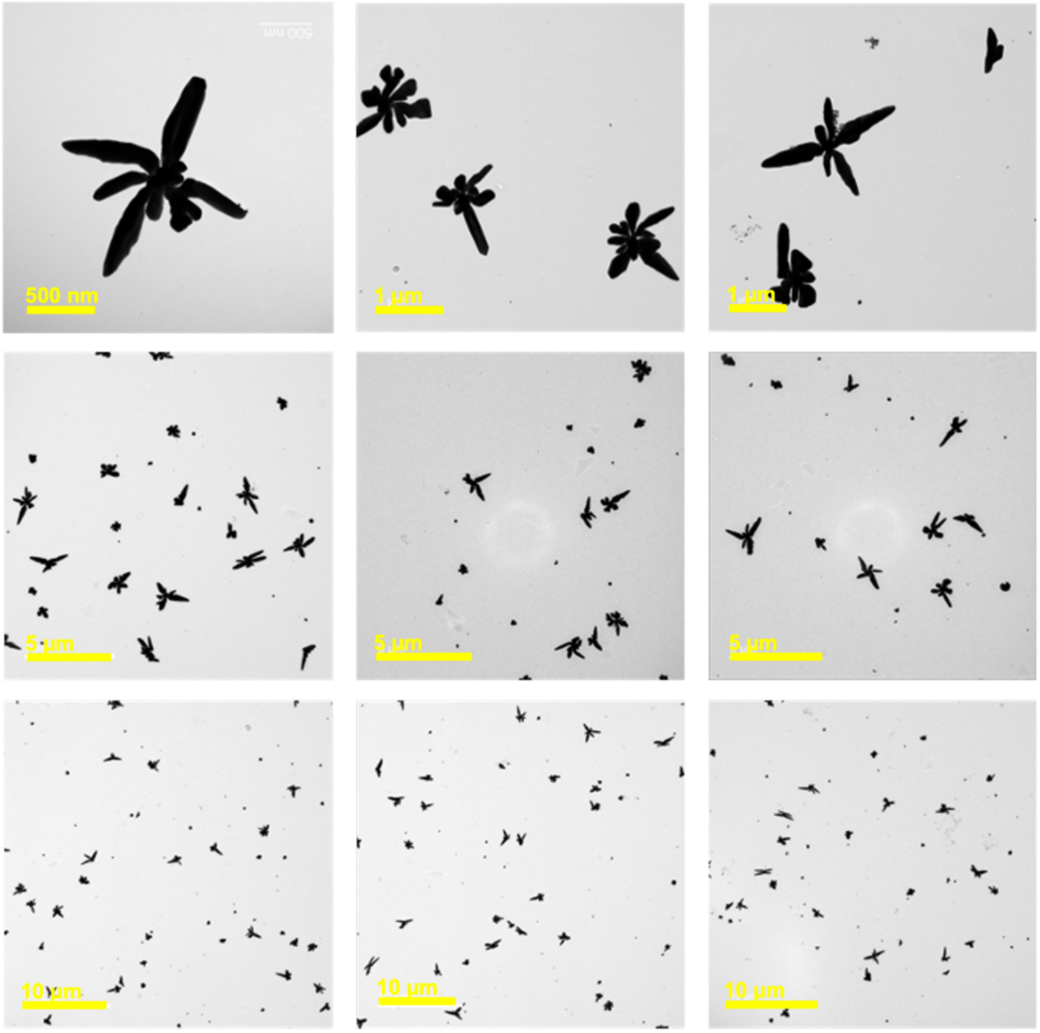

Figure S23. TEM images of BSPP-AgNPs after the addition of $(KKK)_2$ at multiple magnifications.

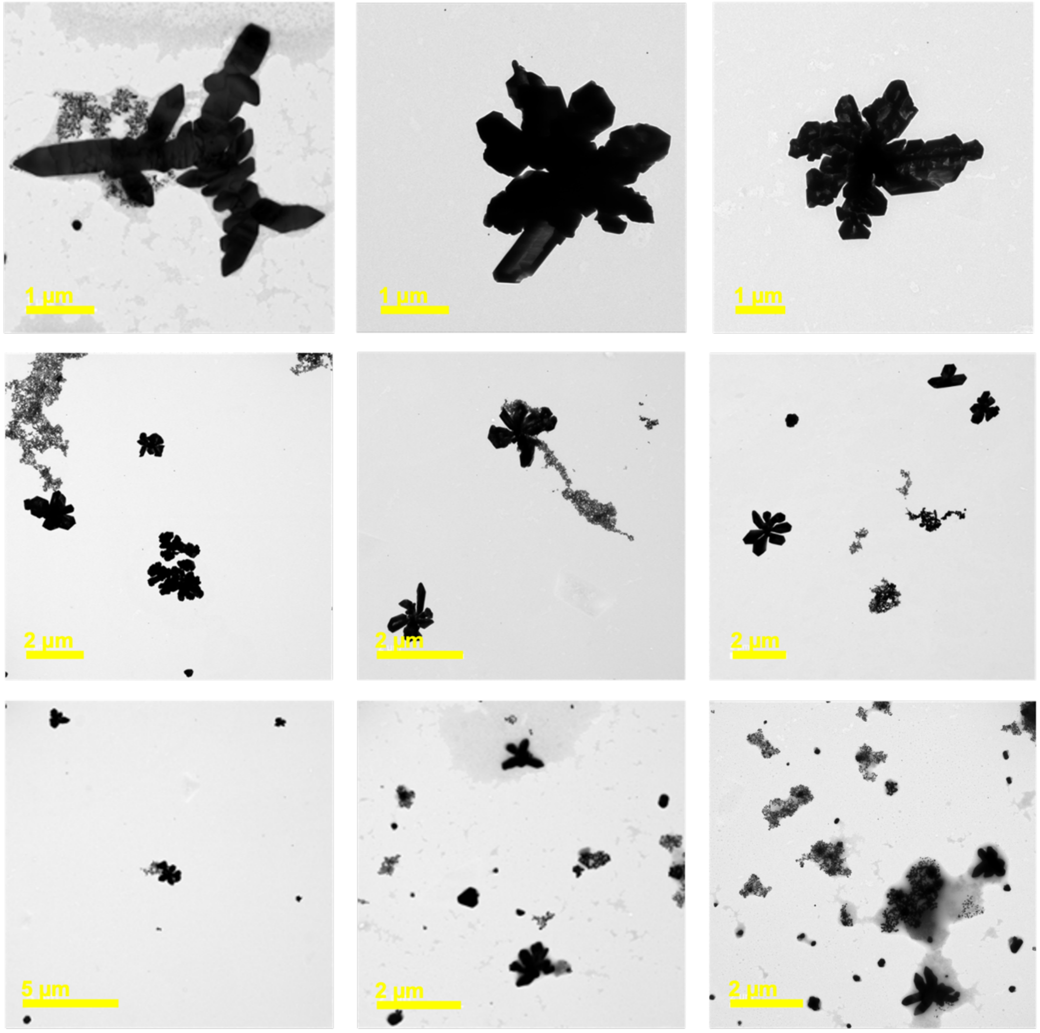

Figure S24. TEM images of BSPP-AgNPs after the addition of $(KKK)_3$ at multiple magnifications.

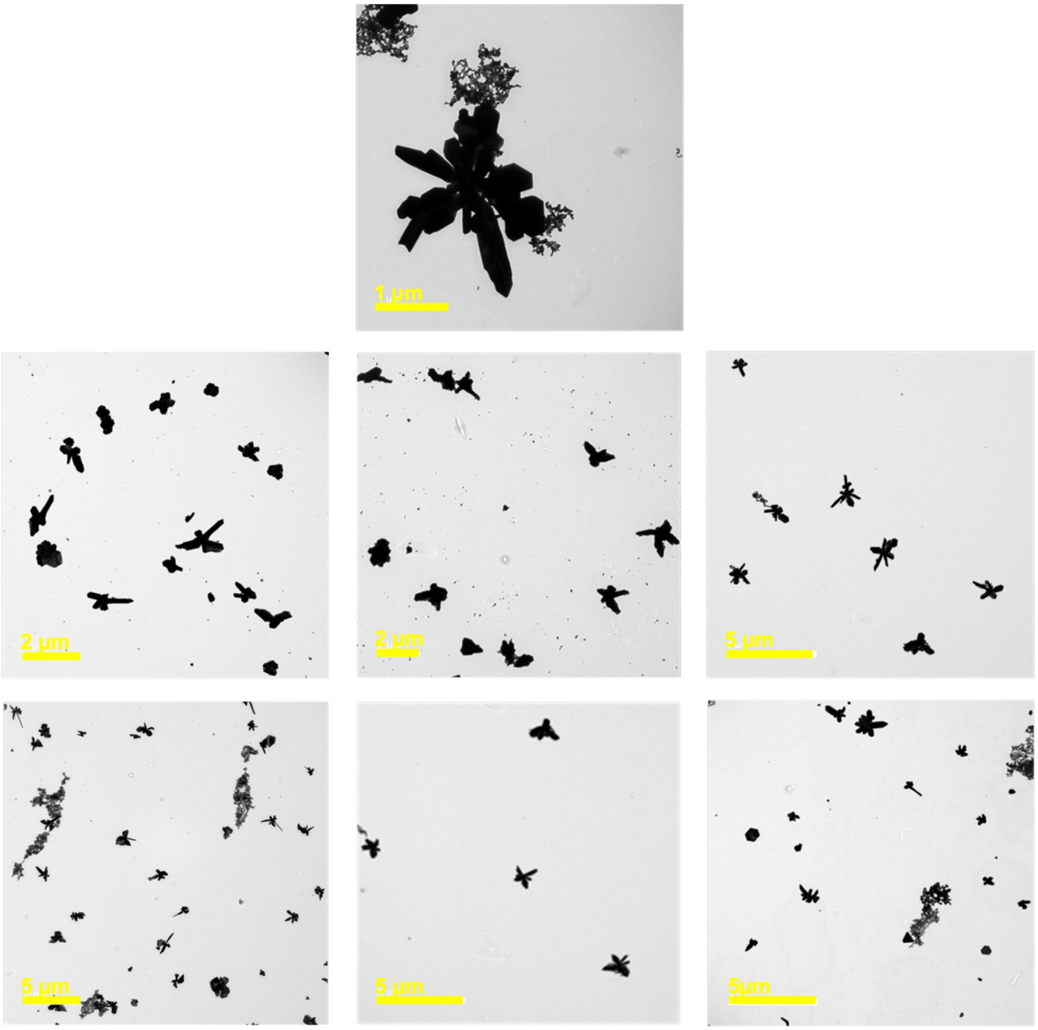

Figure S25. TEM images of BSPP-AgNPs after the addition of HHK at multiple magnifications.

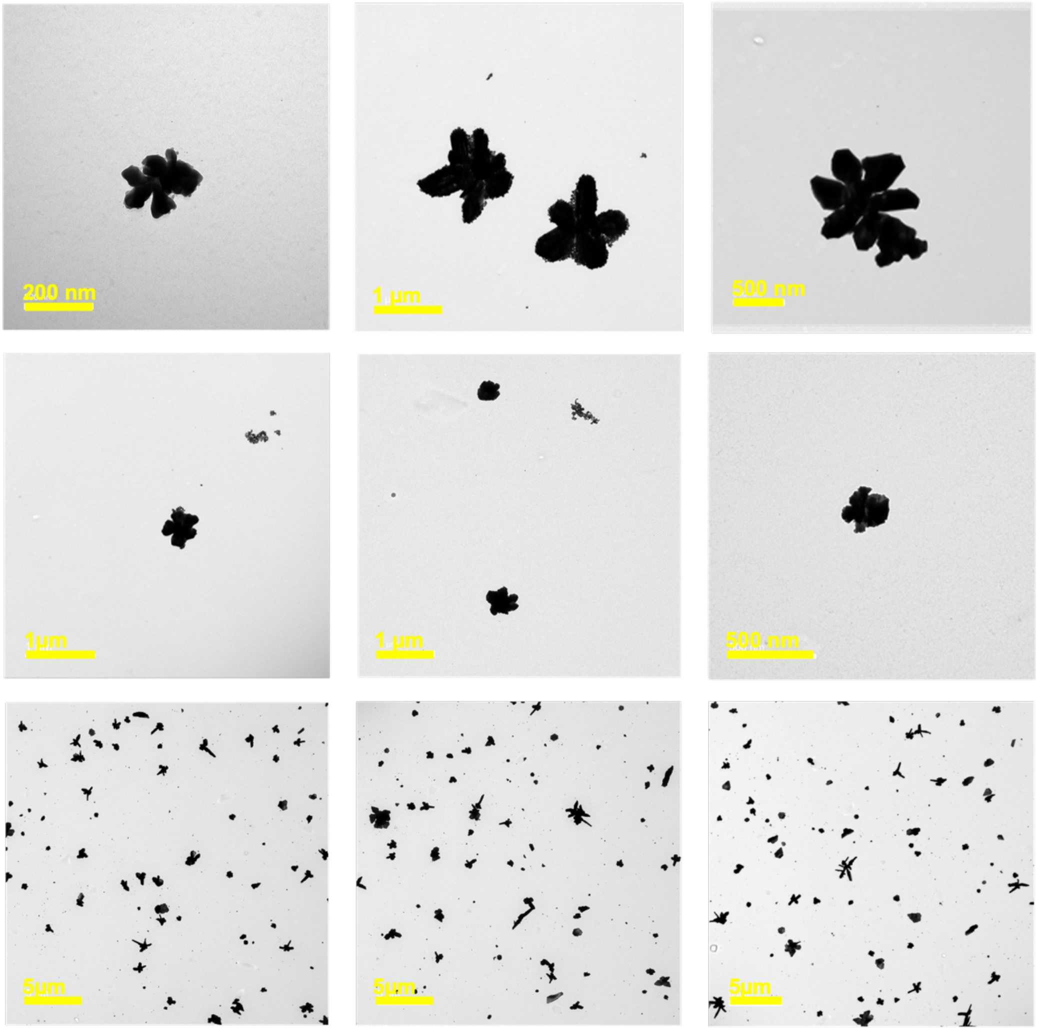

Figure S26. TEM images of BSPP-AgNPs after the addition of $(HHK)_2$ at multiple magnifications.

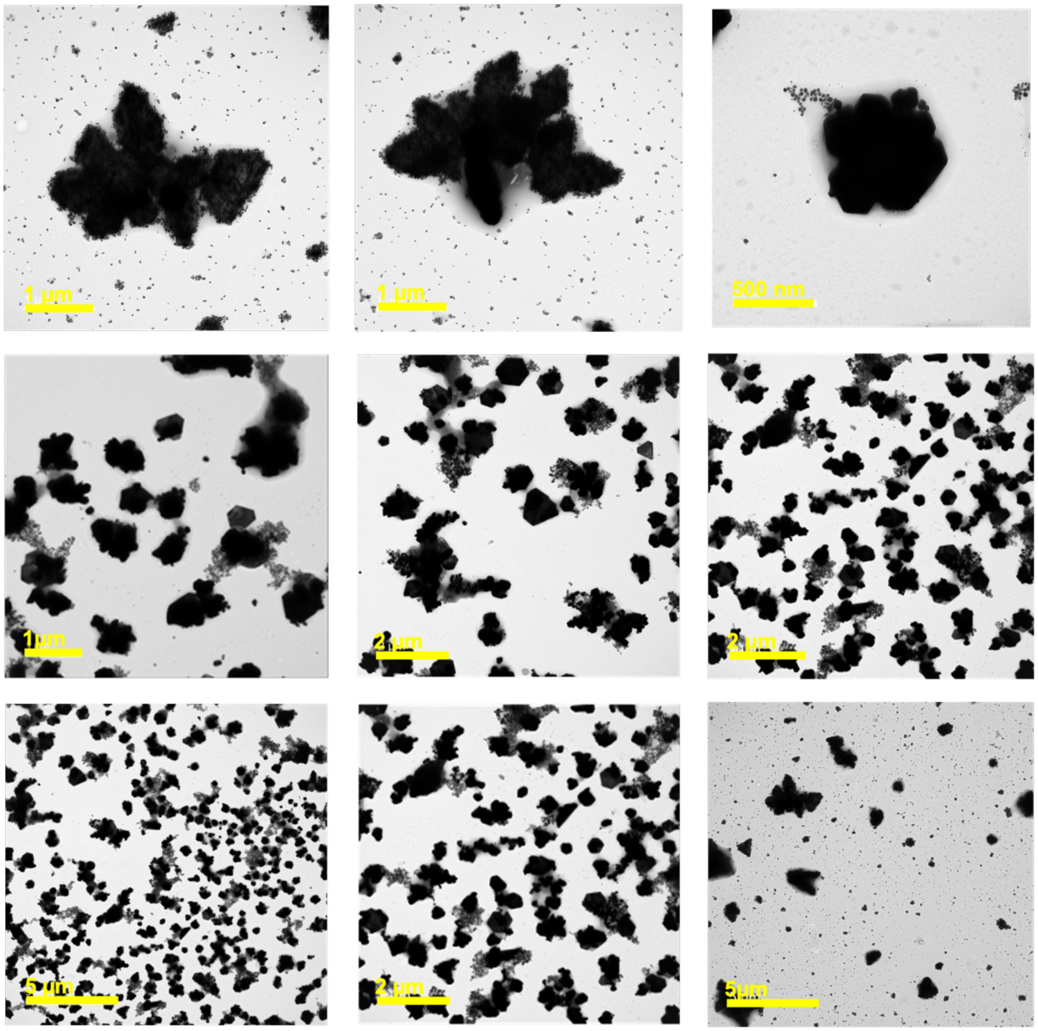

Figure S27. TEM images of BSPP-AgNPs after the addition of $(HHK)_3$ at multiple magnifications.

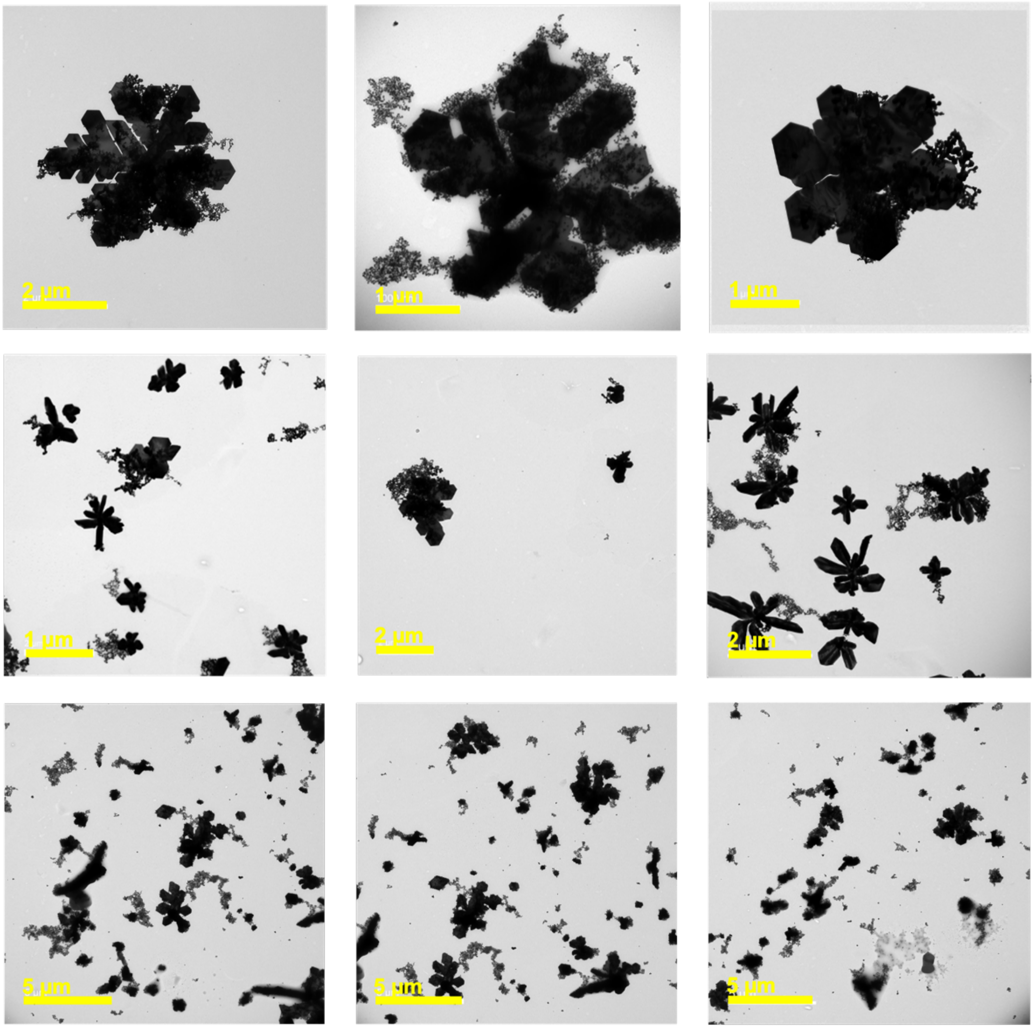

Figure S28. TEM images of BSPP-AgNPs after the addition of WWK at multiple magnifications.

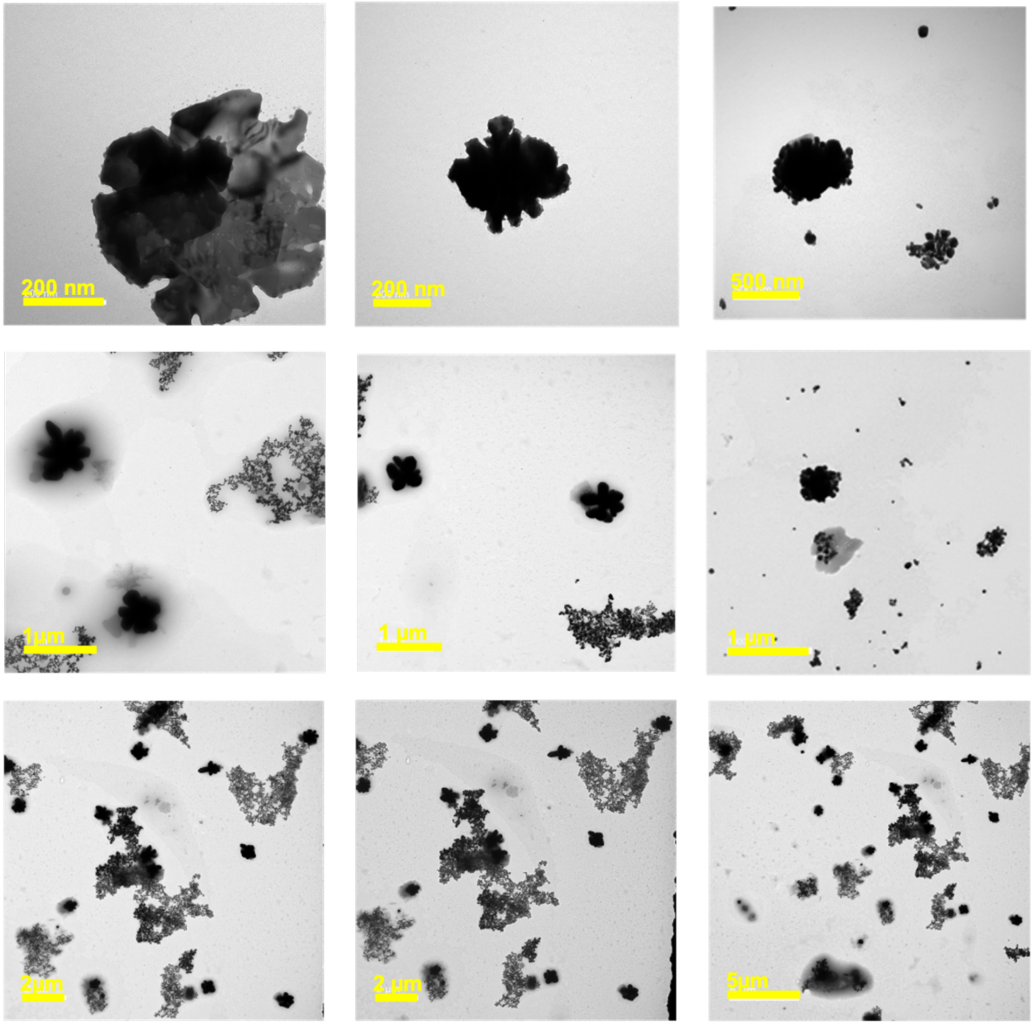

Figure S29. TEM images of BSPP-AgNPs after the addition of $(WWK)_2$ at multiple magnifications.

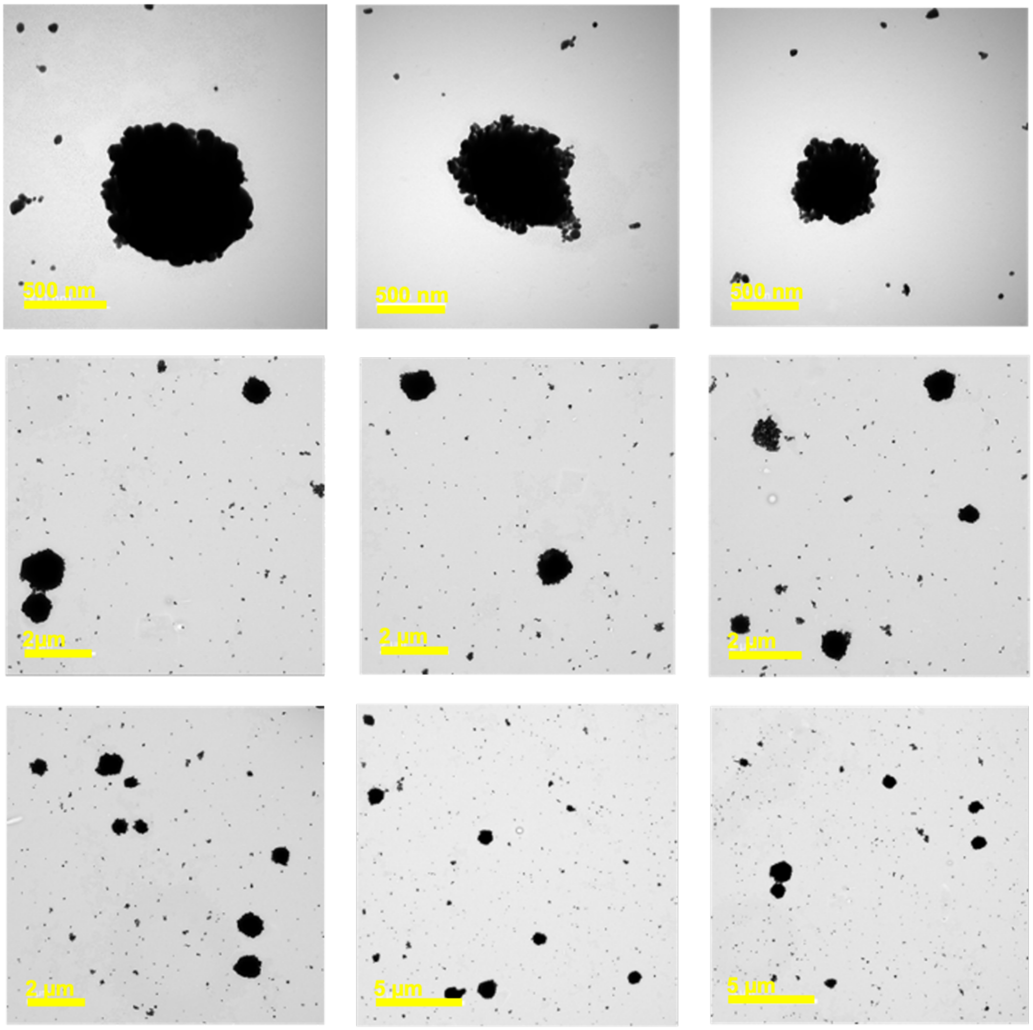

Figure S30. TEM images of BSPP-AgNPs after the addition of $(WWK)_3$ at multiple magnifications.

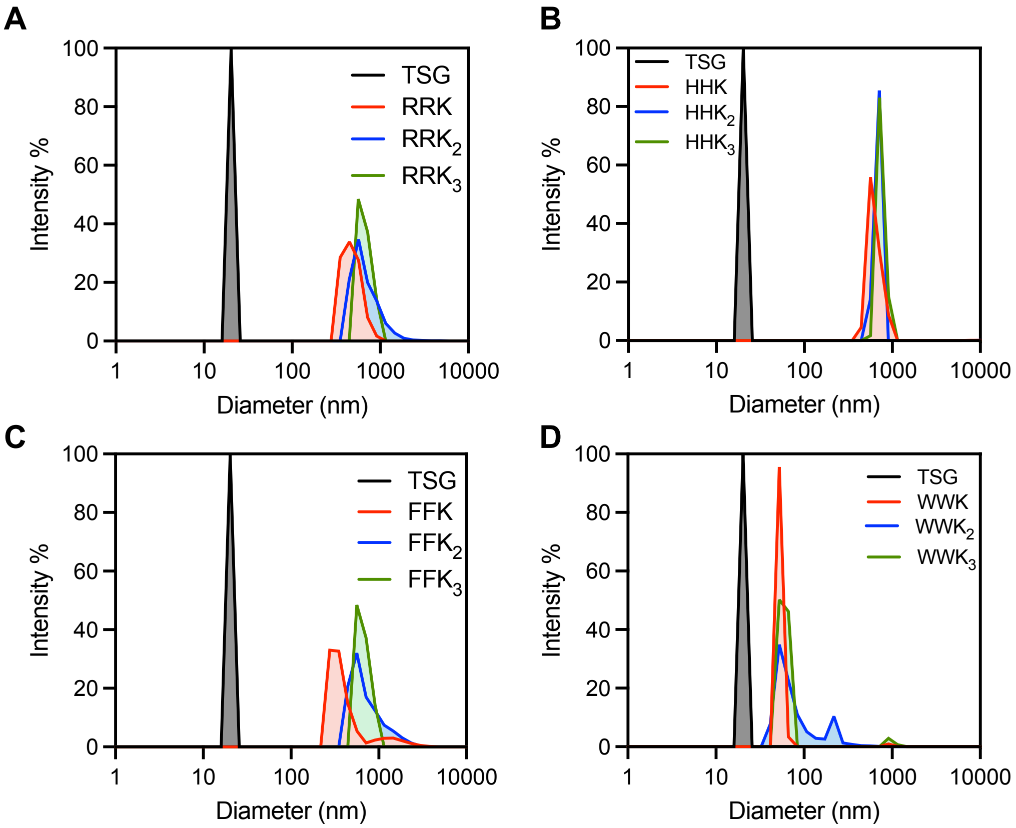

Figure S31. Hydrodynamic diameter (D_H) distribution obtained from dynamic light scattering show an increase in size (~ 1000 nm) proportional to the increase in bridging peptide repetitions. **(A)** Arginine-based peptide family. **(B)** Histidine-based peptide family. **(C)** Phenylalanine-based peptide family. **(D)** Tryptophan-based peptide family.

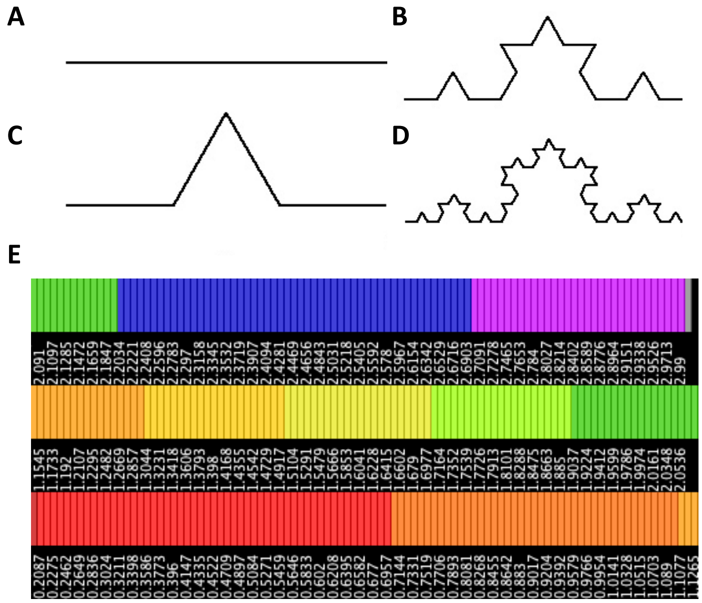

Figure S32. Fractal dimension analysis standard and ROYGBIV color scheme with corresponding fractal dimensions. **(A)** Standard edge for fractal dimension of 0. **(B)** Standard edge for fractal dimension of 1. **(C)** Standard edge for fractal dimension of 2. **(D)** Standard edge for fractal dimension of 3. **(E)** ROYGBIV color scheme corresponding to fractal dimension value.

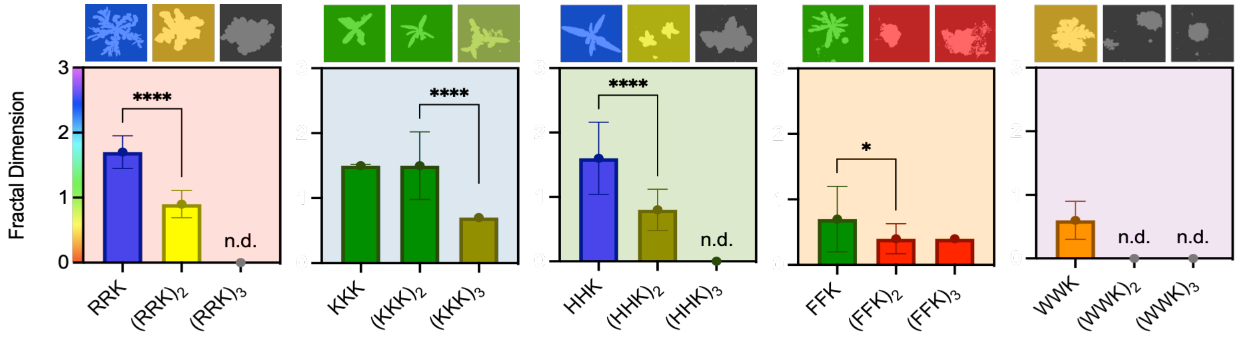

Figure S33. Fractal dimension analysis sample output and statistical analysis summary. Statistical analysis was conducted using GraphPad Prism 10. P-value of < 0.05 was considered significant. Multiple Unpaired Student's t tests were also performed to compare the significance between variables. Error bars represent standard deviation from 24 individually imaged structures. Significance is determined when α < 0.05: **** (p < 0.0001); * (p < 0.05). Not determined: n.d., is denoted when no fractal structure is found by the FracLac extension.

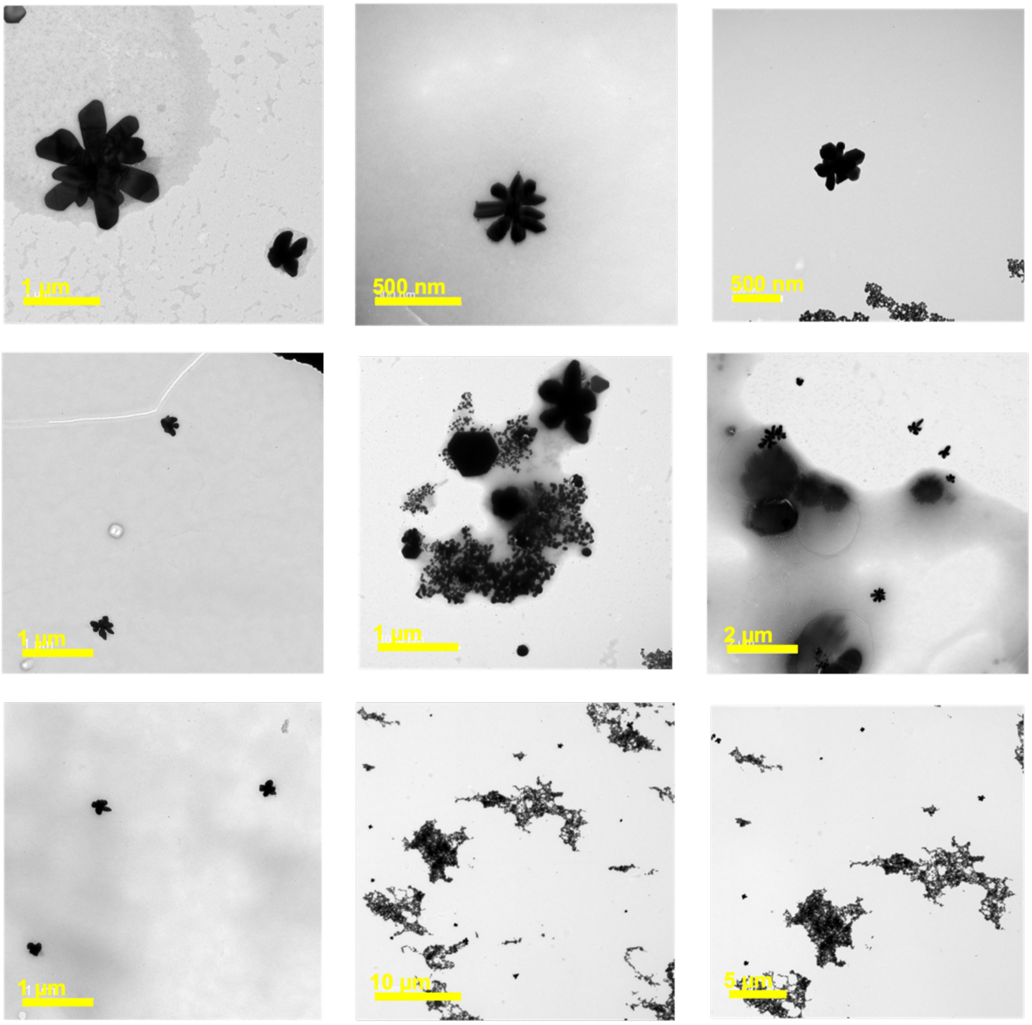

Figure S34. TEM images of BSPP-AgNPs after the addition of 1 µM of RTSGR at multiple magnifications.

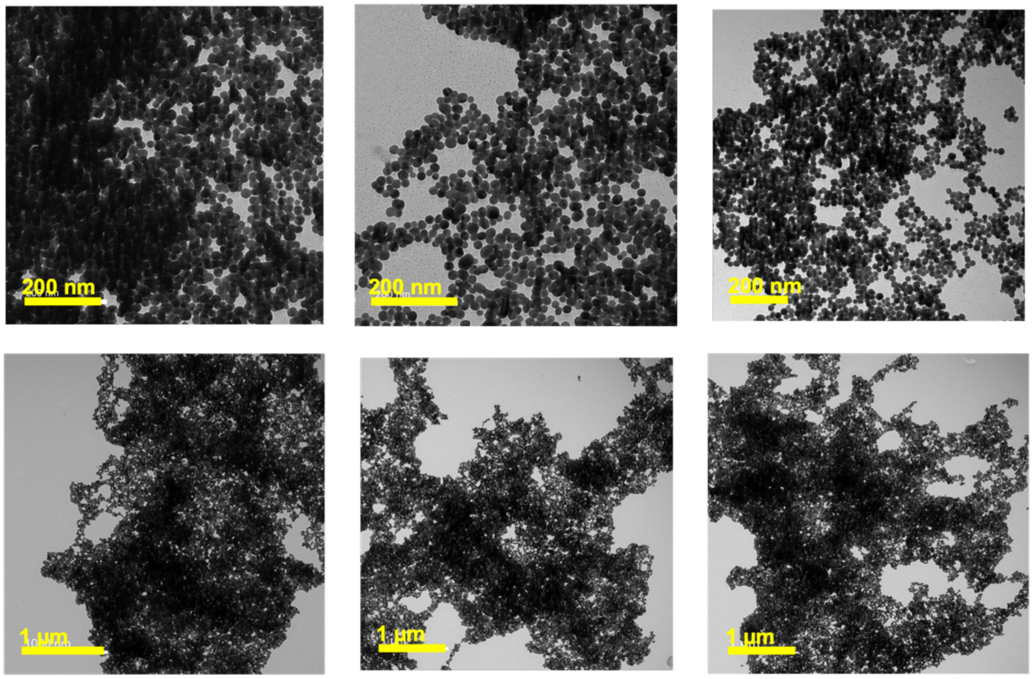

Figure S35. TEM images of BSPP-AgNPs after the addition of 1 µM of R(TSG)$_2$R at multiple magnifications.

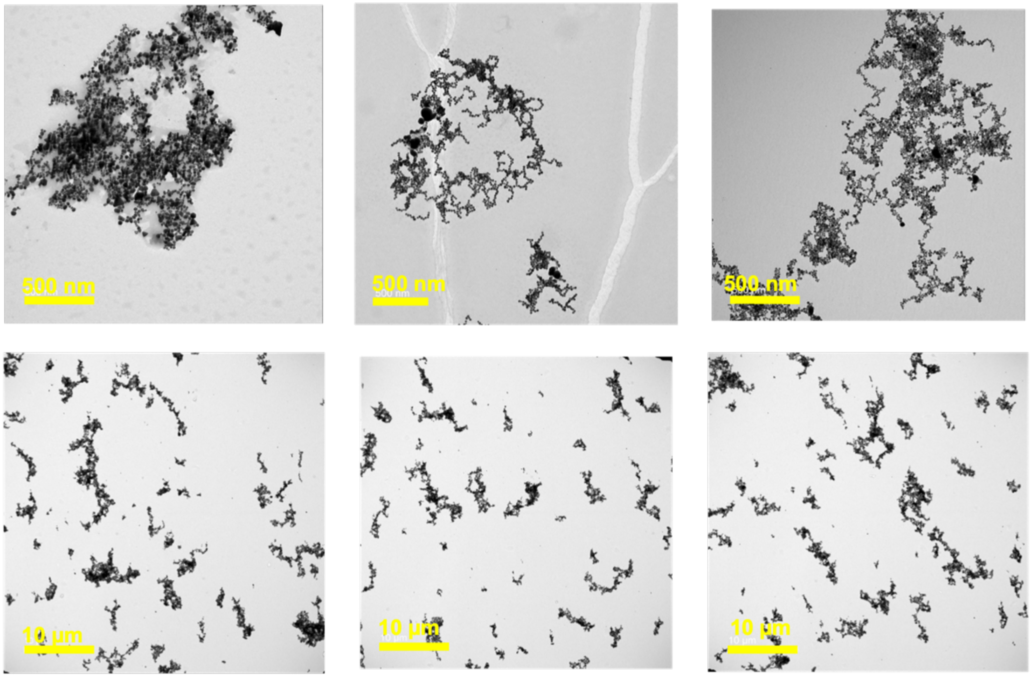

Figure S36. TEM images of BSPP-AgNPs after the addition of 100 µM of R(TSG)$_3$R at multiple magnifications.

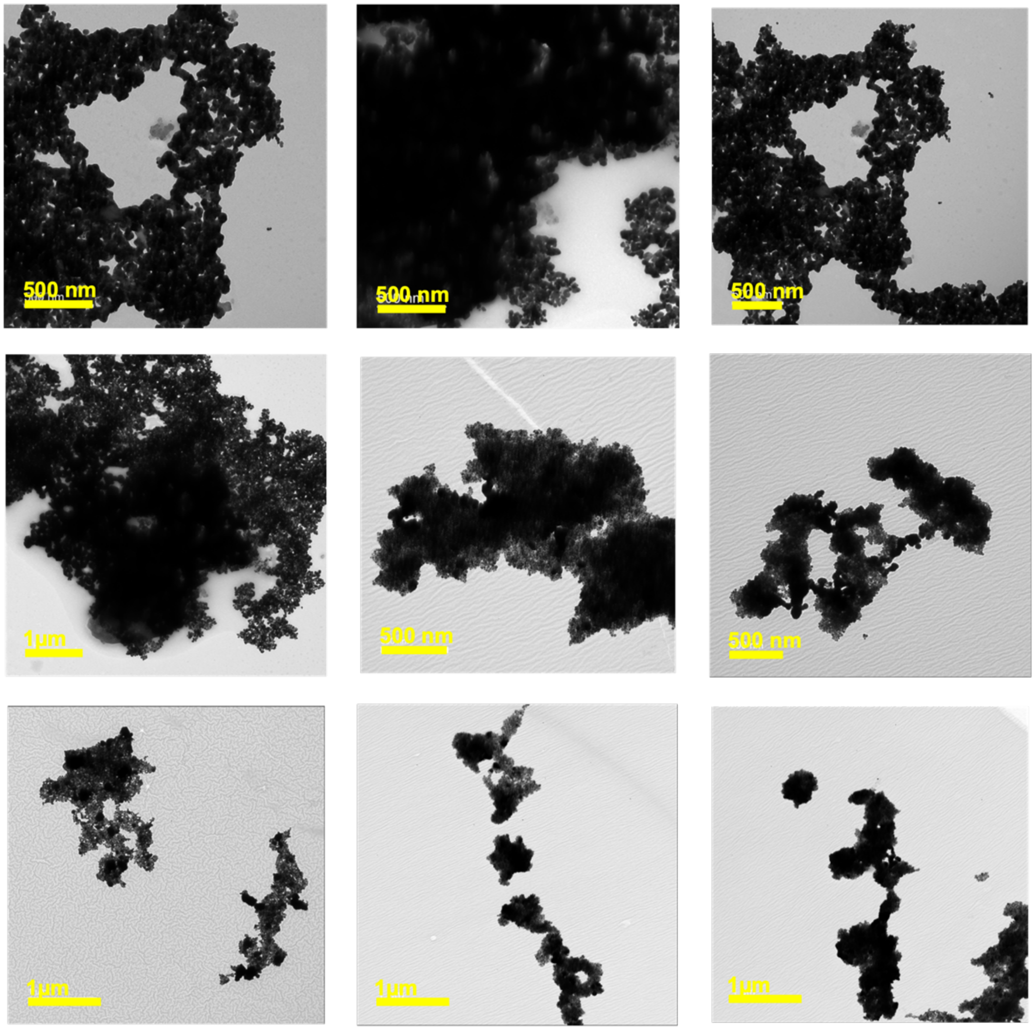

Figure S37. TEM images of BSPP-AgNPs after the addition of 50 μM of RRTSGRR at multiple magnifications.

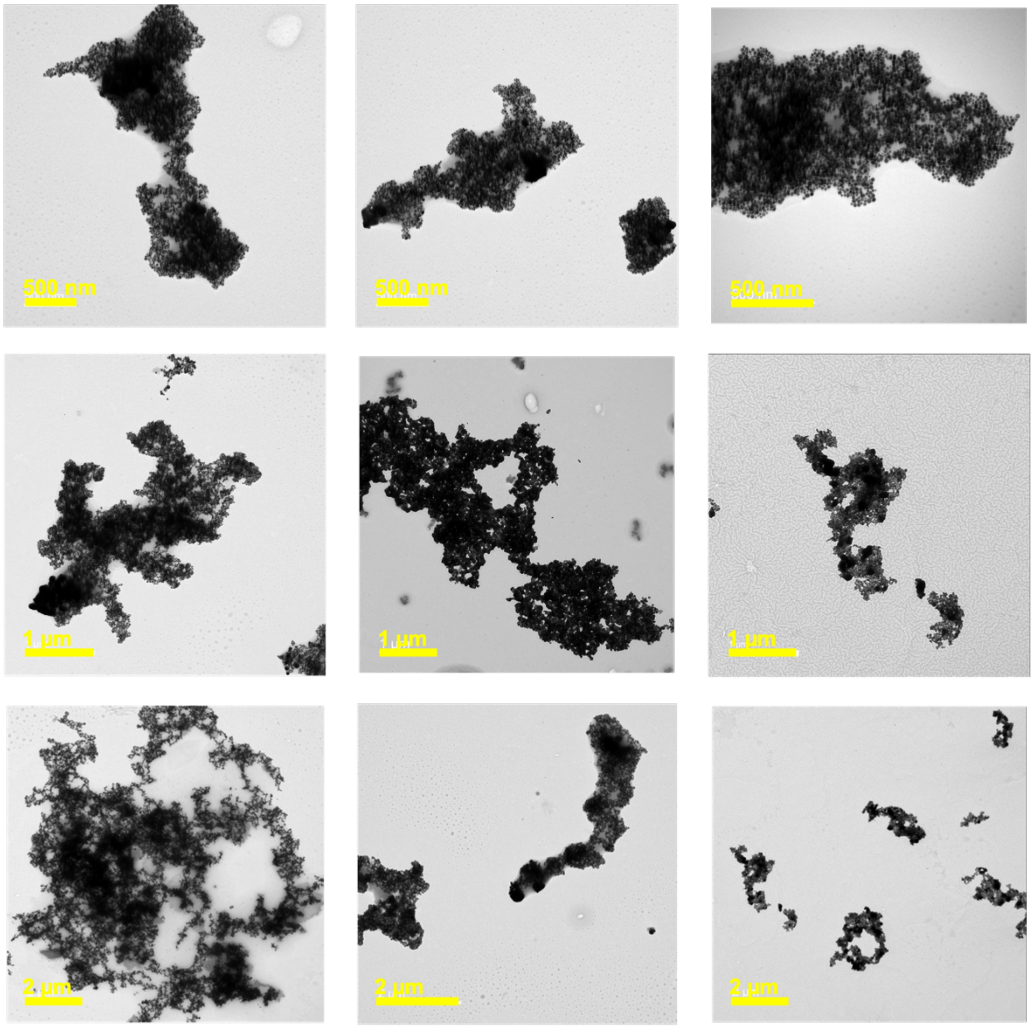

Figure S38. TEM images of BSPP-AgNPs after the addition of 50 µM of $RR(TSG)_2RR$ at multiple magnifications.

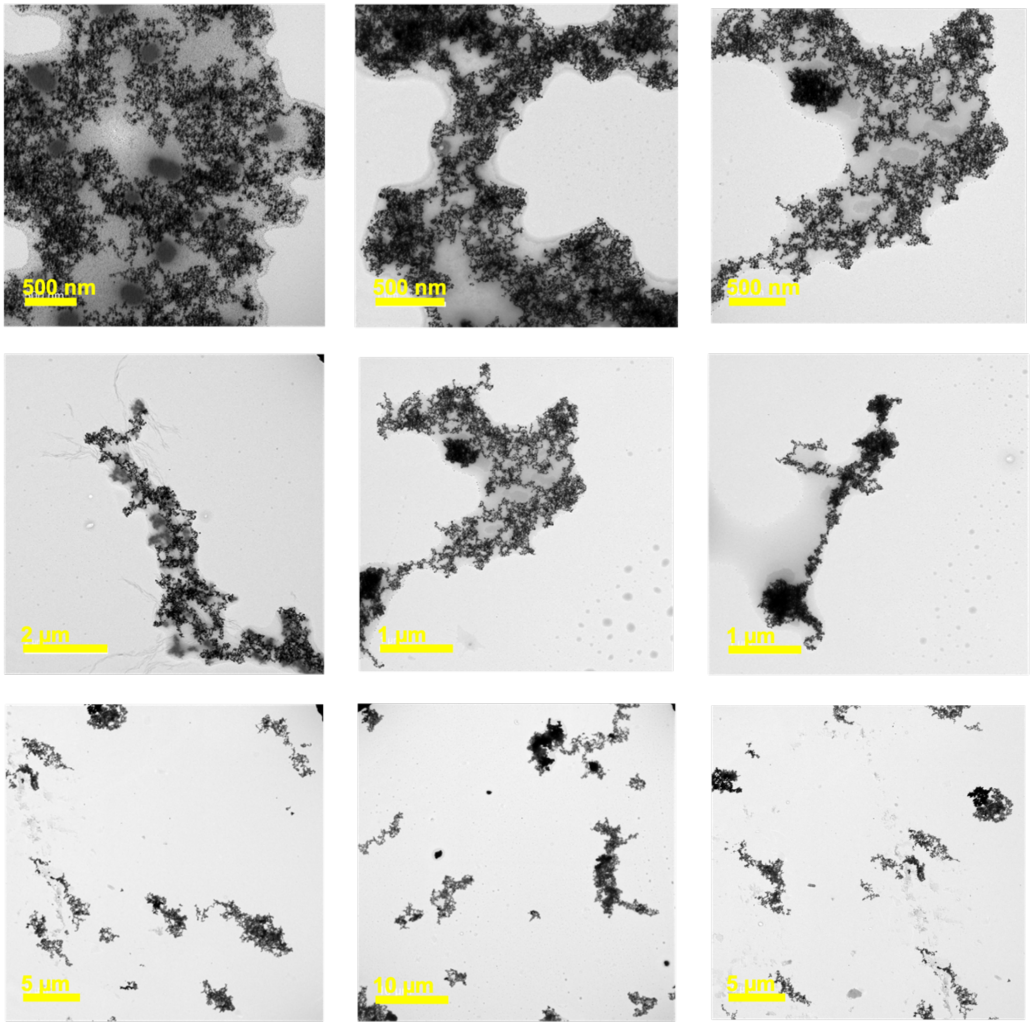

Figure S39. TEM images of BSPP-AgNPs after the addition of 100 µM of RR(TSG)$_3$RR at multiple magnifications.

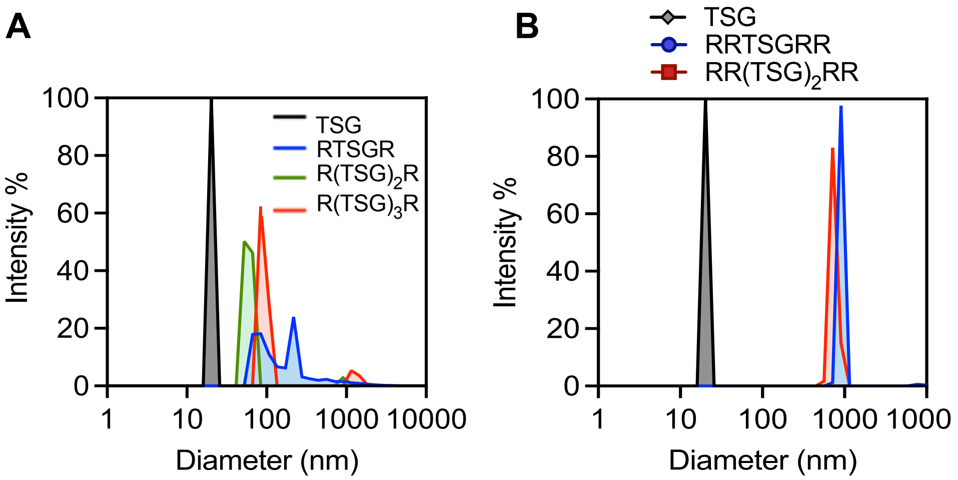

Figure S40. DLS for nonbridging amino acid spacer peptides. **(A)** RTSGR (blue), R(TSG)$_2$R (green) and R(TSG)$_3$R (red). **(B)** RRTSGRR (blue), RR(TSG)$_2$RR (red).

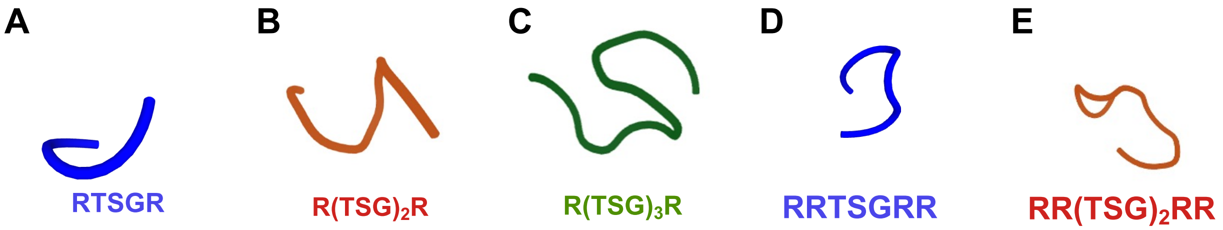

Figure S41. PepFold modeling for nonbridging amino acid spacer peptides. **(A)** RTSGR, **(B)** R(TSG)$_2$R, **(C)** R(TSG)$_3$R, **(D)** RRTSGRR, **(E)** RR(TSG)$_2$RR. PEP-FOLD operates based on a de novo approach for structure prediction using a physics-based energy function to calculate the energetically favorable conformations of the peptide chain in water. This involves considering various forces and interactions, such as bond angles, dihedral angles, and nonbonded interactions. PEP-FOLD generates a large number of potential conformations or structures for the given peptide sequence. This is often done using a Monte Carlo or molecular dynamics sampling approach. Each generated conformation is assigned a score based on its energy and agreement with experimental or theoretical constraints. The algorithm selects the most energetically favorable conformations as potential predictions for the 3D structure of the peptide. Finally, PEP-FOLD provides the user with the predicted 3D structure(s) of the input peptide sequence. Notably, the accuracy of structure prediction tools can vary depending on the length and complexity of the peptide sequence.

IV. Author Contributions

L.A. and J.V.J. conceived the idea. L.A. designed and performed major material syntheses and experimental works. M.R. helped develop methodology for colorimetric detection and interpret microscopy data. Z.J. helped with S/TEM diffraction measurements and interpretation. S.K. assisted with peptide synthesis and characterization. L.A. drafted the manuscript with input from all authors.
